# Group Sex Events Among Cisgender Men Who Have Sex With Men: Cross-Sectional and Longitudinal Survey Study to Explore Participation and Risk-Taking Behaviors

**DOI:** 10.2196/15426

**Published:** 2019-11-27

**Authors:** Lauren R Violette, Lisa A Niemann, Vanessa M McMahan, David A Katz, Pollyanna R Chavez, Hollie A Clark, Andy Cornelius-Hudson, Steven F Ethridge, Sarah J McDougal, George Ure II, Joanne D Stekler, Kevin P Delaney

**Affiliations:** 1 Department of Medicine University of Washington Seattle, WA United States; 2 HIV/STD Program Public Health - Seattle & King County Seattle, WA United States; 3 Division of HIV/AIDS Prevention Centers for Disease Control and Prevention Atlanta, GA United States; 4 Department of Global Health University of Washington Seattle, WA United States; 5 Department of Epidemiology University of Washington Seattle, WA United States

**Keywords:** men who have sex with men, group sex, HIV infection, sexual networks, risk, threesomes, four-or-more-somes

## Abstract

**Background:**

Group sex events (GSEs) are common among cisgender men who have sex with men (MSM), pose a unique risk profile for HIV and sexually transmitted disease (STD) transmission, and may be on the rise, in part because of Web-based networking platforms. However, collecting data on GSEs can be challenging, and many gaps exist in our knowledge about GSE participation among MSM.

**Objective:**

The objective of this study was to develop survey questions addressing aggregate and partner-specific group sex behaviors to measure prevalence of GSEs and associated risks in persons participating in Project Diagnostic Evaluation To Expand Critical Testing Technologies (DETECT), including MSM seeking HIV and STD testing at a public clinic in Seattle, Washington.

**Methods:**

We developed a computer self-assisted survey that included questions about participant demographics, sexual history, and risk behaviors, including group sex, as a part of Project DETECT, a Centers for Disease Control and Prevention–funded study evaluating point-of-care HIV tests. Aggregate and partner-specific questions asked about participation in all GSEs, threesomes, and four-or-more-somes including questions about number and HIV status of sex partners and condom use during the events. To evaluate question performance, we assessed the discrepancies in reporting between the aggregate and partner-specific questions, quantified question refusal rates, and calculated the additional time required to answer the GSE questions. Information about network density (number of partnerships of overlapping duration) was estimated and compared for MSM who did and did not report GSEs.

**Results:**

Among 841 visits by 690 MSM who were asked any group sex survey question, participation in a GSE of any type in the past 3 months was reported at 293 visits (293/841, 34.8%). We found that 9.0% (76/841) of MSM in the sample reported ≥1 four-or-more-some in the partner-specific questions but did not report in the aggregate. The proportion of refusals on any given aggregate GSE-related question ranged from 0% (0/273) to 10.6% (15/141) (median 2.6%) and partner-specific questions ranged from 0% (0/143) to 22% (5/23) (median 3.0%), with questions about four-or-more-somes having the highest proportions of refusals. Completing the aggregate group sex questions added 1 to 2 minutes and the partner-specific questions added an additional 2 to 4 minutes per partner to the total survey length. As expected, the partner-specific GSE questions documented higher density of sexual networks that was not captured by asking about total partner counts and overlap of specific partnerships.

**Conclusions:**

We found that the Project DETECT survey was able to obtain nuanced information about GSEs. The question skip patterns and consistency checks were effective, and survey fatigue was minimal. More research is needed on GSEs, and our survey represents a promising data collection tool to help fill gaps in knowledge about the subject.

## Introduction

A growing body of literature indicates that group sex, a sexual interaction involving more than 2 participants, is common among cisgender men who have sex with men (MSM) [1-6]. These events can vary widely in form and context, including the number of participants, the relationships between participants, the type of sex that occurs, and the site or setting in which the event takes place [1-5]. Prevalence estimates of group sex event (GSE) participation from previous studies among cisgender MSM have ranged from approximately one-quarter of respondents reporting group sex during the last year in a venue-based sample in Washington, DC, to nearly three-quarters of respondents reporting a GSE in their lifetime in an online survey from the London metropolitan area [2,6-10]. GSE participation among cisgender MSM may be increasing, in part because of the proliferation of Web-based and mobile social networking platforms used to facilitate meeting sex partners [6,11,12].

GSEs pose a unique set of risks for transmission of HIV and other sexually transmitted diseases (STDs) because the single event enables STD transmission from 1 infected individual to multiple partners [4,13] more efficiently than a sequence of monogamous events with the same number of partners [14,15]. Some studies have also shown that GSE participation is associated with a higher prevalence of behavior associated with risk for HIV acquisition such as condomless receptive anal intercourse and using drugs or alcohol [7,8,10,12,16-18]. However, others have found that MSM who participated in GSEs were more likely to use condoms or pre-exposure prophylaxis (PrEP) than those who did not participate in GSEs [9,19,20].

Much remains to be learned about the prevalence of GSEs and the behaviors of persons who participate in them. Obtaining nuanced data on GSEs is difficult for many reasons: the sensitivity of the subject may make researchers and participants hesitant to discuss the topic, in-depth questions about GSEs can be complicated and difficult for respondents to navigate, and a history of sexual stigma against MSM might make participants hesitant to report GSE participation [5,21]. Studies on GSEs to date have largely focused on determining prevalence and associated population-level behavioral factors rather than looking at partner-level or event-level data, and the absence of partner-centric questions leaves significant gaps in our ability to understand HIV and STD transmission dynamics and characterize risk associated with GSEs [22]. Partner-specific data are important for network-based models that are increasingly being used in the analyses of HIV and STD transmission because the density and structure of sexual networks have been shown to be important characteristics in mathematical models, especially for MSM [22]. However, the importance of collecting these details needs to be balanced against the additional time and effort required of study participants to provide information on GSE participation. Finding this balance can be particularly challenging in longer national surveys such as the Centers for Disease Control and Prevention (CDC) National HIV Behavioral Surveillance (NHBS) and the American Men’s Internet Survey (AMIS). As part of an ongoing study funded by the CDC to compare the performance of point-of-care HIV tests, we developed a series of aggregate and partner-specific questions to determine the prevalence of GSEs among MSM and characterize condom use and seroadaptive behaviors of participants during GSEs. In this paper, we focus on the development of the survey questions; detailed analysis of survey results will be presented elsewhere.

## Methods

### Study Design and Recruitment

These questions were implemented as part of a larger behavioral survey administered via computer-assisted self-interview (CASI) to participants in Project Diagnostic Evaluation To Expand Critical Testing Technologies (DETECT) [23,24]. Participants were English-speaking individuals aged ≥18 years and either (1) cisgender men and transgender or gender nonconforming individuals who had sex with men seeking HIV testing at the Public Health - Seattle & King County (PHSKC) STD clinic or (2) persons with known HIV infection referred from various sources including the Madison Clinic at Harborview Medical Center (a Ryan White–funded HIV care clinic) and PHSKC HIV/STD Program staff. Participants with negative results from all study HIV tests were able to re-enroll in the study every 90 days. The study was approved by the University of Washington Human Subjects Division (study number 00001637). All participants gave either written or verbal consent (using an identical institutional review board–approved information statement) and were compensated US $40 for study participation. Project DETECT began enrollment in September 2015, and the behavioral survey was piloted from October 30, 2015, to February 23, 2016, with the inclusion of only those participants who had discordant HIV test results (at least 1 positive and 1 negative HIV test result); 4 subjects completed the survey during this time. Beginning February 24, 2016, all study participants, regardless of their HIV test results, completed the survey.

#### Questionnaire

The CASI (Questionnaire Development System [QDS], version 3.0; Nova Research Company) assessed demographics, HIV testing history and interaction with the health care system, current symptoms of acute HIV infection, recent STD history, PrEP use, antiretroviral treatment (ART) use, substance use, and sexual behaviors at the aggregate and partner-specific levels. Participants were asked between 18 and 287 questions, depending on their responses. A blank piece of paper was provided to help participants with recall and track details during survey completion. Study staff were available to provide clarifications regarding questions if requested. Participants were able to refuse all questions in the survey other than the documentations for study consent and specimen storage, sex at birth, current gender, and gender of sex partners, if any, in the last year.

#### Analysis Sample

This analysis was limited to a subset of cisgender MSM who participated in Project DETECT. We defined MSM as reported male sex at birth, current male gender identity, and anal sex with at least 1 man in the past 3 months. These participants were first asked if they participated in any GSEs and then about threesomes and four-or-more-somes separately. Although GSEs are generally defined in the literature as a sexual interaction between ≥3 people, participants might consider *group sex* only to include 4 or more participants [4]. Distinguishing between threesomes and four-or-more-somes is potentially important because participation in threesomes may be associated with different partnership characteristics and behaviors than participation in four-or-more-somes [1]. For the purposes of this paper, we refer to GSEs to encompass all events that involve ≥3 persons including the study participant, threesomes to include events with 3 people, and four-or-more-somes to indicate GSEs that included ≥4 people.

#### Development of Group Sex Questions

As there are no pre-existing validated measures, the group sex questions were modeled on NHBS and AMIS, in which participants are asked about sexual behaviors in aggregate and then asked about behaviors with their most recent partner(s) [25,26]. When the last reported sex occurred in the context of a GSE, such questions about *most recent partner* are challenging to answer because of simultaneous partnerships. We, therefore, modified these questions for Project DETECT to account for the possibility of GSEs in the previous 3 months by asking several questions about group sex participation with the participant’s 3 most recent anal sex partners.

#### Aggregate Group Sex Questions

Depending on responses to previous questions and preprogramed skip patterns, participants were asked between 1 and 76 questions about involvement in threesomes and four-or-more-somes in the previous 3 months (Multimedia Appendix 1).

**Figure 1:**
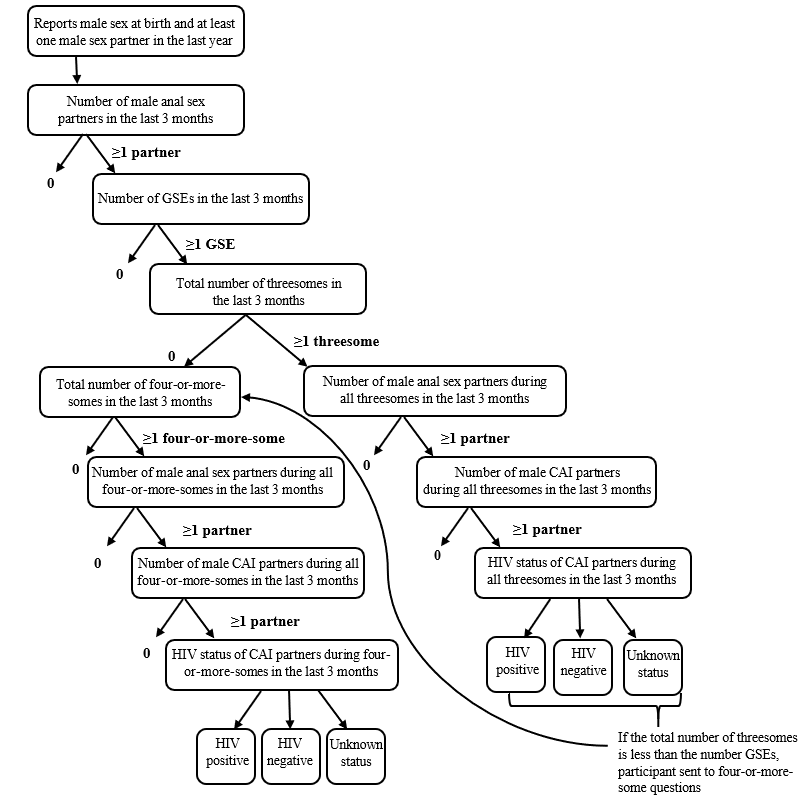
Questions and skip patterns for aggregate group sex questions. CAI: condomless anal intercourse; GSE: group sex event.

Figure 1 illustrates the flow of the aggregate group sex questions. Participants who reported having ≥1 GSEs in the past 3 months were asked to report the number of threesomes during that time (Figure 1). Participants who reported at least 1 threesome were asked about the total number of anal sex partners across all threesomes; total number of partners with whom they had condomless anal intercourse (CAI) across all threesomes; and distribution of CAI partners who were HIV-positive, HIV-negative, or whose HIV status was unknown. Participants were then asked the same series of questions about four-or-more-somes if the number of reported threesomes was less than the number of total reported GSEs. If a participant refused to provide an answer to a question, they were skipped to the next appropriate question. Participants were not able to select “don’t know” as an answer option to any of the aggregate GSE questions.

Consistency checks were programed within the aggregate threesome and four-or-more-some questions to ensure data accuracy. The total number of threesomes plus four-or-more-somes could not be greater than the total number of reported GSEs. Participants were not able to report more anal sex partners or CAI partners within GSEs than the total number of anal or CAI partners they had reported earlier in the survey. The number of CAI partners in threesomes plus four-or-more-somes could not exceed the number of anal sex partners reported across all GSEs. Finally, the sum of HIV-positive, HIV-negative, and unknown status CAI partners in GSEs was required to equal the total number of CAI partners reported in GSEs. From these data, the overall percentage of MSM reporting anal sex in the past 3 months who also reported participating in group sex can be estimated. Several other measures, such as the percentage of all male sex partners who were group sex partners as well as subgroups (eg, men who reported group sex but only threesomes) can also be calculated.

#### Partner-Level Group Sex Questions

Partner-level data were collected to describe up to 3 of the participants’ most recent anal sex partners (partners A-C) during the past 3 months. Participants were asked a series of questions about their relationship and sexual behaviors with partners A to C. Questions included partner demographics, when and how the participant met the partner, the self-defined nature of their relationship (eg, “is/was partner A-C someone that you feel or felt committed to?”), partner STD history, HIV status disclosure, partner HIV status and ART or PrEP use, number of sexual encounters with that partner in the past 3 months, and the dates of first and most recent anal sex encounters with the partner (Multimedia Appendix 1). We also asked about concurrency between partners A to C (ie, sexual relationships with different partners in the same period) and whether the participant knew if partners A to C had sex with each other in the same time frame that they were having sex with the participant, also referred to in network modeling literature as a nondirected transitive triad or a known triangle [27]. Those who reported ≥3 anal sex partners in the past 3 months were also asked aggregate questions to describe those additional partners, including the nature of their relationship, whether they engaged in CAI in the past 3 months, and the HIV status of partners with whom they had CAI.

**Figure 2:**
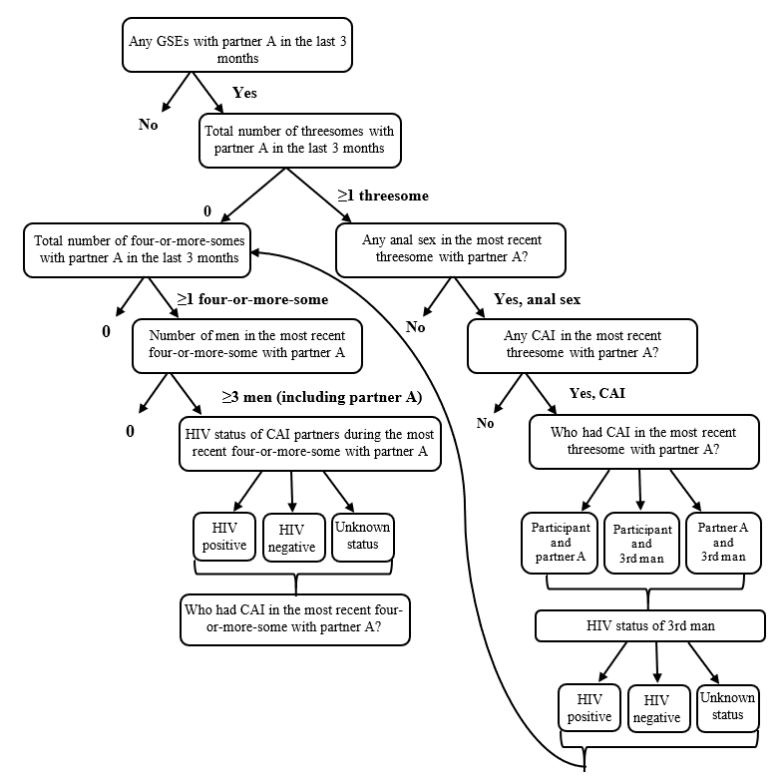
Questions and skip patterns for partner-specific group sex questions. CAI: condomless anal intercourse; GSE: group sex event.

If the participant reported anal sex with any of partners A to C, they were asked if they had any threesomes or four-or-more-somes in the last 3 months that involved that partner. If so, they were asked to provide additional details about the most recent GSE involving that partner (Figure 2). Participants were asked about the number of threesomes that involved the partner, whether any anal sex and CAI occurred (between any participants of the threesome) in the most recent threesome involving the partner, who had CAI within the most recent threesome, and the HIV status of the third man in the most recent threesome with the partner. Participants were then asked the number of four-or-more-somes in the past 3 months involving the partner. If participants reported at least 1 four-or-more-some with any of partners A to C, they were asked about the number of men in the most recent four-or-more-some with that partner.

**Figure 3:**
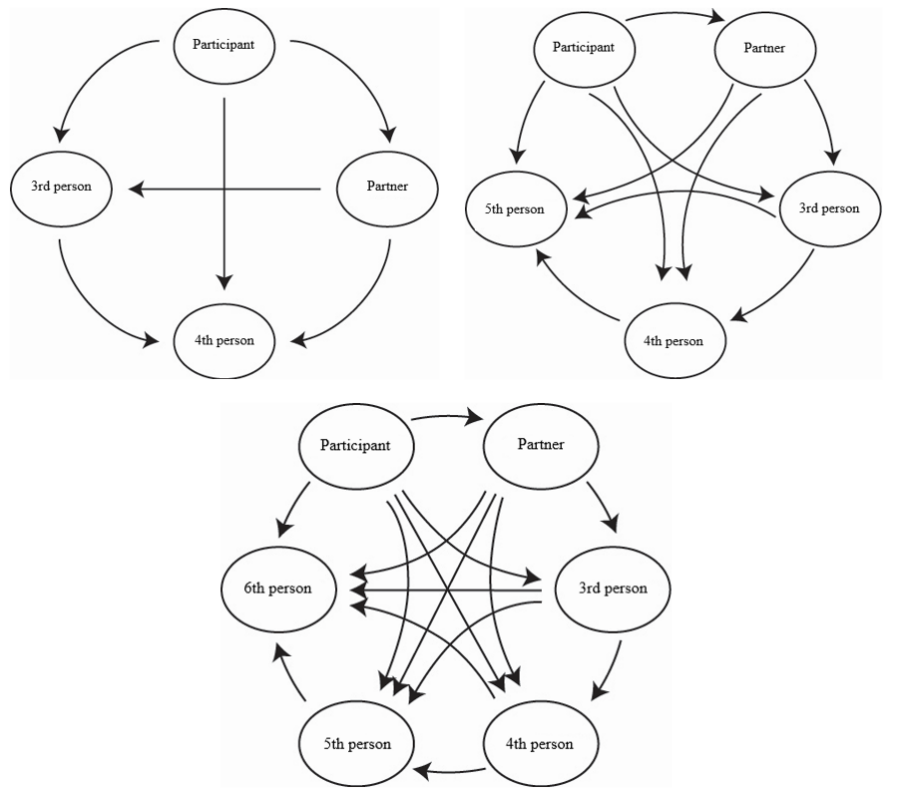
Increasing complexity of partner-partner interactions with increasing size of group sex events.

Although we asked for the HIV status of all men in the participant’s most recent four-or-more-some, questions about condom use between each set of partners were limited to 4 people (partner A plus 3 additional people in the GSE) to minimize participant burden. Figure 3 demonstrates the rapidly increasing numbers of possible partner combinations with each additional partner in the GSE. For example, a threesome includes 3 potential partner-pair interactions, whereas a six-some includes 15 (Figure 3). Due to programing limitations within the survey software, we were unable to include a table or figure to simultaneously display possible partner combinations for the participant, which might have been more intuitive for some participants to complete. Therefore, each question was programed as an individual screen containing 1 question about condom use during the most recent event between 2 individuals (Multimedia Appendix 1). Participants were able to select “don’t know” when asked about the HIV status of partners, including the third person in their most recent threesome with partners A to C. Selecting “don’t know” was also allowed when asked about CAI between sets of partners that did not include the participant during the most recent four-or-more-some.

We programed consistency checks within the partner-level GSE questions. For participants reporting sex with any named partners A to C and at least 1 other man in the same encounter, responses should have reflected at least 1 threesome or four-or-more-some with that partner; if not, the survey would return to the overall GSE question for that specific named partner to reconcile this inconsistency. The number of partners in any reported four-or-more-some was required to be 3 or more. In addition, the sum of HIV-positive, HIV-negative, and unknown status partners in the most recent four-or-more-some with any of the named partners A to C was checked to ensure that it was equal to the total number of individual participants reported in the GSE.

#### Data Accuracy and Consistency Between Aggregate and Partner-Level Group Sex Questions

Owing to concern for participant burden, there were no programed consistency checks for accuracy between subtotals of GSEs reported in the aggregate and in the partner-specific sections. Participants may, therefore, report a different aggregate total of threesomes or four-or-more-somes when compared with the reported number of threesomes or four-or-more-somes involving partners A to C. This could include reporting no GSE participation in the aggregate questions and then reporting GSE activity with any partners A to C. If we had included such consistency checks and an inconsistency occurred between the aggregate and partner-specific questions, participants would have been directed back to the aggregate questions to check their previous responses. This would have required returning to questions much earlier in the survey and reanswering multiple questions, potentially including all 3 sets of partner-specific questions. For the purpose of the descriptive analysis and comparison of participation in GSEs by visit (described below), any reported GSE activity in either the aggregate or the partner-specific question sets by a participant was identified as GSE participation. Discrepancies between the aggregate and partner-level responses were also explored during analysis.

### Statistical Analysis

We first aimed to determine the acceptability and feasibility of the group sex questions by assessing participants’ willingness to respond to them, survey fatigue and added burden related to these questions, and the consistency of participants’ responses within and across study visits. We calculated the overall proportion of refusals in the aggregate and partner-specific GSE question sets and compared refusal proportions for questions about partner A against questions about partner C to assess completeness of data collection and to determine if there was survey fatigue with more question exposure for participants.

We also compared the length of time required to complete the survey based on the start and end times. We compared the distribution of total time to complete the survey for those with 2 to 3 anal sex partners and those with ≥4 partners, as participants who reported ≥4 anal sex partners received up to 20 additional questions about their relationship with those partners. We then stratified by whether or not the participant reported aggregate or partner-specific group sex to assess how much time the different group sex questions added to the overall survey duration.

To assess consistency of responses within a single survey, we examined discrepancies between GSE participation reported in the aggregate and partner-specific questions of the survey and calculated frequencies of GSE reports for each question set. We also created a composite measure of group sex participation that combined any GSE report in either the aggregate or partner-specific question set and then used the composite to report the frequency of group sex participation in the sample.

To assess consistent reporting of GSEs among participants who enrolled in the study more than once, we used a person-specific medical record number to identify repeat enrollments and compared reported GSEs across all visits for each individual person.

After assessing these data quality and survey-taking measures, we summarized the additional information about network density gleaned from the group sex questions, compared with asking only aggregate or partner-specific questions about one-on-one partnerships. We calculated 4 measures of network density of partners with whom the participant reported CAI: (1) mean degree, that is, number of CAI partners in the past 3 months; (2) the percentage of participants reporting partner concurrency, that is, relationships with 2 different men in which the participant reported having CAI with both men in the same 3-month period; (3) known triangles, that is, the percentage of named partners A to C with whom the participant reported both having CAI and knowing that the 2 partners had CAI with each other; and (4) additional and otherwise hidden triangles and higher-order partner overlap, that is, the percentage of participants who did not report that named partners A to C had sex with each other in the past 3 months but did report that they had a GSE with the partner in which both the participant and that partner had CAI with at least 1 additional man.

In this last measure, we used questions about the most recent GSE to quantify occurrence of CAI with up to 3 additional partners besides the participant and the named partner A to C, which would not have been observed without the addition of the partner-specific group sex questions.

We compared these 4 measures among MSM with at least 2 total partners (meaning they could have engaged in group sex) and at least 1 anal sex partner in the previous 3 months (which was the subset of participants who were asked the GSE and partner-specific survey questions), stratified by whether or not they reported a GSE. We used Wilcoxon rank sum test to compare means and chi-square tests to compare percentages. All analyses were conducted using SAS version 9.4 (SAS Institute Inc).

## Results

From September 2015 to September 2017, there were 1260 study visits in Project DETECT. Of the 833 HIV-negative study participants, 163 repeat participants (163/833, 19.6%) had a median of 2 (interquartile range [IQR] 2-3; range 2-7) study visits. Behavioral surveys were completed by 854 individual participants at 1104 visits. Behavioral surveys were missing for 154 individual participants across 156 study visits. Among study visits with a completed survey, male sex at birth was reported during 1071 (1071/1104, 97.01%) visits, and of those, 1038 (1038/1071, 96.92%) visits were among participants who identified as male gender. Of these 1038 visits, participants reported at least 1 male anal sex partner in the past 3 months (MSM) and were, therefore, asked at least 1 of the GSE questions at 841 (841/1038, 81.02%) visits with 690 individual people.

Table 1 shows GSE participation as reported through the aggregate and/or partner-specific GSE questions. In 293 (293/841, 34.8%) visits, participants reported a GSE of any type in the previous 3 months; at least 1 threesome or four-or-more-some in the past 3 months was reported in 270 (270/841, 32.1%) and 137 (137/841, 16.3%) visits, respectively (categories not mutually exclusive). For each reported GSE activity, we found that some participants did not report GSE participation in the aggregate question set but did report at least 1 GSE event with partner(s) A, B, and/or C (Table 1). Notably, 76 of 137 (55.5%) four-or-more-somes were reported by MSM in the partner-specific question set but were not reported in the aggregate. In addition to whether or not any GSEs were reported, there were also discrepancies in the number of threesomes and four-or-more-somes; for all types of GSEs, there were instances where participants reported a higher number of events in the partner-specific section when compared with their response in the aggregate (data not shown).

**Table 1:** Comparison of reported group sex event participation in aggregate and partner-specific group sex event questions among men who have sex with men (N=841).

Type of GSE^a^	Reported in either aggregate or partner-specific questions or both, n (%)	Reported in both aggregate questions and partner-specific questions, n (%)	Reported in aggregate questions but not partner-specific questions, n (%)	Reported in partner-specific questions but not aggregate questions, n (%)
Reported GSE(s)	293 (34.8)	191 (22.7)	82 (9.8)	20 (2.4)
Reported threesome	270 (32.1)	168 (20.0)	81 (9.6)	21 (2.5)
Reported four-or-more-some	137 (16.3)	29 (3.4)	32 (3.8)	76 (9.0)

^a^GSE: group sex event.

Among the 841 visits with MSM where GSE questions were asked, 261 visits were with 110 individual people who enrolled in the study multiple times. Approximately half of the 110 repeat participants reported no GSEs at any of their study visits, whereas 20 of 110 (18.2%) reported GSEs at every visit where they were asked the questions. The remaining 37 repeat participants (37/110, 33.6%) reported GSEs at 1 or more, but not all, of their study visits.

The proportions of refusals to aggregate-level GSE questions ranged from 0% (0/273) to 10.6% (15/141), with a median of 2.6% (Table 2), and to partner-level questions stratified by partner ranged from 0% (0/143) to 22% (5/23), with a median of 3.0% (data not shown). The proportion of refusals to questions about partner C was higher when compared with that about partner A (data not shown).

**Table 2:** Refusal rates of aggregate group sex event questions.

Aggregate GSE^a^ question	Was asked question (n)	Refused to answer question (n)	Proportion refused (%)
Number of times participant had a GSE	841	9	1.1
Number of times participant had a threesome	273	0	0
Number of men participant had anal sex with during all threesomes	249	4	1.6
Number of men participant had CAI^b^ with during all threesomes	211	1	0.5
Number of CAI partners during threesomes whose HIV status was unknown	169	10	5.9
Number of CAI partners during threesomes who were HIV-positive	169	13	7.7
Number of CAI partners during threesomes who were HIV-negative	169	13	7.7
Number of times participant had a four-or-more-some	141	15	10.6
Number of men participant had anal sex with during all four-or-more-somes	61	0	0
Number of men participant had CAI with during all four-or-more-somes	54	0	0
Number of CAI partners during four-or-more-somes whose HIV status was unknown	39	1	3
Number of CAI partners during four-or-more-somes who were HIV-positive	39	3	8
Number of CAI partners during four-or-more-somes who were HIV-negative	39	3	8

^a^GSE: group sex event.

^b^CAI: condomless anal intercourse.

The time required to complete the survey increased with both overall partner number and the number of partners with whom the participant reported group sex (Table 3). For participants reporting 2 to 3 total anal sex partners, the survey took a median of 18 minutes if they did not report group sex or only reported group sex in the aggregate questions. Participants asked the additional partner-specific group sex questions took 1 to 2 additional minutes per partner to complete the survey. Those with ≥4 partners were asked an additional set of questions about the characteristics of those partners; on average, this group took an additional 3 minutes regardless of group sex participation. Those with ≥4 partners who reported partner-specific GSEs with all 3 of partners A to C took between 8 to 10 additional minutes to complete the version of the survey with all the additional partner-specific survey questions.

**Table 3:** Time required to complete the Project Diagnostic Evaluation To Expand Critical Testing Technologies (DETECT) behavioral survey, stratified by total number of anal sex partners and the number of partners with whom the participant reported participating in group sex in the previous 3 months.

Reporting of group sex in aggregate and partner-specific questions	Reported 2-3 anal sex partners		Reported ≥4 anal sex partners	
	n	Median (IQR) minutes	n	Median (IQR) minutes

No group sex reported in the aggregate and no partner-specific group sex reported	233	18 (14-22)	122	21 (17-27)
Group sex reported in the aggregate but no partner-specific group sex reported	20	18 (14-26)	55	20 (17-28)
Group sex reported in the aggregate, partner-specific group sex with 1 partner	46	20 (16-35)	63	24 (20-31)
Group sex reported in the aggregate, partner-specific group sex with 2 partners	22	21 (19-27)	44	26 (20-36)
Group sex reported in the aggregate, partner-specific group sex with 3 partners	4	21 (19-25)	20	28 (23-33)

Table 4 subsets the sample further by limiting to MSM who reported at least 2 male sex partners, the number of persons needed for a GSE to occur. Data in Table 4 illustrates both the types of network density data collected through the partner-specific and aggregate group sex questions and how these questions impact information available about the structure of MSM sexual networks for those who do and do not report group sex. Men who reported participating in group sex reported a higher mean number of CAI partners in the past 3 months than those with at least 2 partners who did not report group sex participation (4.1 vs 2.0; *P*<.001). Although men who reported group sex had a similar likelihood of any concurrent partnerships compared with participants who did not report GSEs (27.0% vs 23.4%; *P*=.28), they were much more likely to report that named partners also had sex with each other (a triangle) compared with participants who did not report any GSEs (26.0% vs 5.9%; *P*<.001). The aggregate questions that asked who had anal sex with whom during the most recent group sex encounter found that 14.2% of participants who did not report that partners A to C had sex with each other did report a group sex encounter where the participant and 1 of these named partners had CAI with at least 1 additional man.

**Table 4:** Comparison of measures of network density for persons reporting ≥2 sex partners and at least 1 anal sex partner by self-reported group sex event participation in the previous 3 months.

Measures of network density	≥1 group sex event reported (n=289)	No group sex event reported (n=410)	Examples
Mean number of condomless anal intercourse (CAI) partners	4.1	2.0	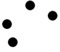
Proportion of participants reporting concurrency between anal sex partners, %	27.0	23.4	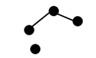
Proportion of participants who reported that ≥1 of named partners A-C had sex with each other (triangles), %	26.0	5.9	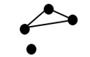
Proportion of participants who reported that named partners A-C did not have sex with each other but the participant, a partner, and ≥1 other person had CAI during the most recent GSE (triangles and higher-order CAI partner overlap), %	14.2	Not applicable	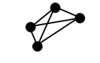

## Discussion

### Principal Findings

In the context of Project DETECT, we developed and tested aggregate and partner-level questions about recent GSE participation among MSM. The questions allowed for effective analysis of nuanced information about overall and partner-specific GSEs. We found that a GSE of some type in the past 3 months was reported in 293 (34.8%) visits with MSM that we surveyed—a finding that is consistent with what has been previously reported in the literature [6,7,10,13,20,28,29].

Owing to discrepancies in reporting GSE participation between the aggregate and the partner-specific question sets, we created a composite measure of the 2 responses to form a more complete estimation of GSE participation in this population. The largest discrepancy between questions was in reporting four-or-more-somes; 9% of MSM responding to these questions reported in the aggregate questions that they did not participate in a four-or-more-some but then later reported at least 1 four-or-more-some with at least 1 of their named partners A to C in the partner-specific section. This demonstrates the importance of asking about GSEs in multiple ways, including in the context of specific partners, to increase the likelihood of recall or question completion. Asking only aggregate GSE questions or partner-specific questions in our sample would have led to underreporting and resulted in missed opportunities for further assessing the role that simultaneous partnerships play in HIV transmission. Studies among MSM have also illustrated the importance of partner- or event-level data in addition to aggregate measures, specifically in the context of CAI [30-33] and substance use [34,35].

Overall, the majority of participants answered the GSE questions completely; the proportion of answer refusals ranged from 0% to 10.6% in the aggregate questions and from 0% to 21.7% in the partner-specific questions. Refusal rates for some of the partner-specific questions were somewhat higher than those for the aggregate questions, which could be because of survey fatigue, sensitivity of the questions asked, or that participants were not able to report specific details about their partners’ behaviors in larger GSEs. Within the partner-specific questions, refusal rates for questions about partner C were higher than those about partner A. It is possible that the partner that participants labeled partner A may be the one they knew most about, were closest to, or had sex with most recently. With this bias, it is not surprising that the refusal rates for questions about partner C are higher than the refusal rates for partner A, although survey fatigue may have also contributed to this difference.

Compared with participants who reported no group sex in either the aggregate or the partner-specific questions, exposure to the aggregate GSE questions did not increase the mean time it took to complete the survey. Those who were also asked the partner-specific questions took an additional 2 to 4 minutes, on average, to complete the survey. Despite the longer survey duration, both sets of group sex questions provided novel information about the density of MSM sexual networks. This extra time spent is relatively minor compared with the overall average survey duration (19 minutes), and these questions are critical to understand the association between GSEs and HIV and STD acquisition as well as to parameterize network models that are being used to estimate the impact of different interventions on HIV epidemics [14,15,22,36].

Approximately one-fifth of the HIV-negative participants re-enrolled in our study and had multiple research visits over the course of the period that we evaluated. Of the 110 repeat participants who answered the group sex questions at multiple study visits, 36.4% reported GSEs at some, but not all, of their study visits, indicating that person-level changes did exist. This illustrates the importance of using data from all visits in future analyses, as restricting to 1 visit per person would have resulted in an underestimate of GSE participation among this sample of MSM.

The implementation of these questions allows us to identify novel information about the density of the sexual networks of MSM. Traditional behavioral surveys have described the number and percentage of all partners that are CAI partners. Only recently have surveys also tried to describe the overlap of partnerships and the duration of partnerships of different types. To our knowledge, this is the first survey that has asked about detailed partner-level interactions, enabling an understanding of the risk-taking behaviors between partners within a GSE and increasing our knowledge of network density. We found that similar amounts of concurrency were reported by those with multiple anal sex partners who did and did not report participation in GSEs. Perhaps not surprisingly, we were also able to document that those who engaged in group sex were much more likely to report knowing that 2 of their recent named sex partners had also had sex with each other. However, our novel partner-specific questions about the most recent group sex encounter found a subset of those who engaged in group sex reported having CAI with several of the men in the encounter. This sharing of sexual partners has been shown to lead to dense subpopulations within the overall sexual network that enhance and sustain the possibility of transmission of STDs. The details of this sexual partner overlap would not have been captured without the additional partner-specific questions specifically about group sex.

### Limitations

There are limitations to this study that should be considered. Results from our study participants in Seattle, Washington, may not be representative of all MSM or of other geographic areas where HIV testing, care for people living with HIV, PrEP, and other services may be less accessible or available. Owing to these differences, it is possible that our participants may be more likely to have GSEs or may have been more willing than others outside of Seattle to report on sensitive information. The majority of participants in this sample were recruited for study participation while seeking HIV testing at a local STD clinic, which means this sample may have different levels of recent HIV risk than MSM recruited from other venues. In addition, these group sex questions were not cognitively tested or validated among this population before study enrollment, which might have impacted the proportion of survey questions that were refused by participants. Anecdotally, no study participants asked clarifying questions to the study research staff, and most of the participants completed the group sex section of the survey.

Our survey asked questions about GSEs only to persons who reported being born male and who had at least 1 male anal sex partner in the past 3 months, and our analysis sample restricted further to only participants who identified as a man at the time of the survey. Participants who identify as something other than a man, MSM who report only oral sex, transgender and genderqueer individuals who do not report male sex at birth, and individuals who report nonmale partners are eligible for Project DETECT, but were not asked the group sex questions. In addition, we asked participants about *male* partners in GSEs but did not specify that partners within GSEs had to be cisgender men, which might have led to misclassification of partners included in the most recent event.

Participants also may have experienced GSEs with transgender, genderqueer, or female partners, but those events were not captured by the current version of our questions. Little research has been done on transgender and genderqueer individuals in the context of group sex, but like cisgender MSM, they are at higher risk for HIV acquisition. A 2017 meta-analysis by Baral et al [37] found an estimated pooled HIV prevalence of 21.7% in transgender women in the United States. Though prevalence estimates in transgender men are lower, the current scope of research is limited [38]. Future surveys assessing group sex participation at the aggregate and partner-specific levels should include these groups in research, as conflating MSM with other genders may not fully address the differences in HIV risk [39].

While assessing these survey questions, we saw that participants did engage in GSEs where no anal sex was reported. In this version of the survey, we only asked about GSEs if the participant reported anal sex in the previous 3 months, thereby missing potentially important information about partner interactions and networks that might include only oral sex.

The partner-specific question set was limited to the 3 most recent male anal sex partners. Although we did collect some information on additional anal sex partners after the 3 most recent, we did not collect the same level of detail (Multimedia Appendix 1). As four-or-more-somes can include those additional partners, collecting more detailed data can add to our knowledge about network density and concurrency.

Finally, because of concern for survey length, the partner-specific questions detail only the most recent threesome and four-or-more-some. A participant’s most recent GSE may not be consistent with other GSEs in the previous 3 months in which they have participated, particularly in this sample of men presenting to an STD clinic seeking an HIV test. However, research among MSM and other populations shows moderate agreement between reports of behaviors at last sex and period-level prevalence questions, indicating that last sex can serve as a valid proxy of behaviors over a period [40,41]. Although collecting detailed information about all GSE participation in the previous 3 months could be beneficial in helping to understand potential HIV and STD transmission, researchers must consider balancing this detail with participant burden and potential recall issues.

### Future Research

The paper describing the analysis of the impact of group sex participation on both STD and HIV acquisition in Project DETECT will be forthcoming. This study has prompted additional related research and modifications to our original protocol. We asked 1 of the GSE questions of the venue-based sample of MSM recruited for NHBS in Seattle in 2017, as well as the 2018 AMIS survey, to field these questions in other populations than the one described here. We believe that our findings illustrate potential for these questions to be incorporated in other national surveys and should be piloted, and ultimately validated, in different geographic regions and with different populations.

Since collecting these data, the Project DETECT behavioral survey has been updated to address some of the limitations described above. As stated above, the survey now collects aggregate GSE information from MSM who report only oral sex in the previous 3 months. This revision will enable us to collect data from MSM who may have only oral sex but be involved in GSEs where CAI is occurring between other partners.

A Spanish language version of the survey has also been created for Project DETECT to include those who can read and write in Spanish but not English (Multimedia Appendix 2). This population is important to include in HIV studies, as HIV diagnoses among Hispanic/Latino MSM increased 13% nationally between 2011 and 2015, and, in King County, where our survey was administered, Latino MSM are 39% more likely than white MSM to have an HIV diagnosis in their lifetime [42,43]. Studies that utilize surveys similar to ours could incorporate our questions to assess responses in additional sites and populations.

Using a survey software that has the ability to include tables or pictorial representations could aid in the ability to collect more accurate and detailed quantitative information about GSEs and interactions that involve multiple simultaneous partnerships. In addition, performing qualitative interviews could help us better understand STD and HIV risk in the context of GSEs, which would improve future survey instruments.

### Conclusions

It is crucial to have appropriate tools to measure and understand GSEs, a sensitive but important topic for the sexual health of MSM. Our study demonstrated that although no one set of questions performed perfectly, implementing a survey with both aggregate and partner-level questions could provide a detailed picture of GSE participation and the density of sexual networks. The questions seemed to be acceptable, skip patterns and consistency checks were effective, and survey fatigue was minimal. More research is needed on this subject, and our survey represents a promising data collection tool to help fill the gaps in our knowledge.

## Appendix

Multimedia Appendix 1 Project Diagnostic Evaluation To Expand Critical Testing Technologies behavioral survey including group sex questions (English).

Multimedia Appendix 2 Project Diagnostic Evaluation To Expand Critical Testing Technologies behavioral survey including group sex questions (Spanish).

## Abbreviations

AMIS: American Men’s Internet Survey
ART: antiretroviral treatment
CAI: condomless anal intercourse
CASI: computer-assisted self-interview
CDC: Centers for Disease Control and Prevention
DETECT: Diagnostic Evaluation To Expand Critical Testing Technologies
GSE: group sex event
IQR: interquartile range
MSM: men who have sex with men
NHBS: National HIV Behavioral Surveillance
PHSKC: Public Health Seattle-King County
PrEP: pre-exposure prophylaxis
QDS: questionnaire development system
STD: sexually transmitted disease

## References

[1] Grov C, Rendina HJ, Ventuneac A, Parsons JT (2013). HIV risk in group sexual encounters: an event-level analysis from a national online survey of MSM in the U.S. J Sex Med.

[2] Gama A, Abecasis A, Pingarilho M, Mendão L, Martins MO, Barros H, Dias S (2017). Cruising venues as a context for HIV risky behavior among men who have sex with men. Arch Sex Behav.

[3] Mimiaga MJ, Reisner SL, Bland SE, Driscoll MA, Cranston K, Isenberg D, VanDerwarker R, Mayer KH (2011). Sex parties among urban MSM: an emerging culture and HIV risk environment. AIDS Behav.

[4] Friedman SR, Mateu-Gelabert P, Sandoval M (2011). Group sex events amongst non-gay drug users: an understudied risk environment. Int J Drug Policy.

[5] Meunier E (2014). No attitude, no standing around: the organization of social and sexual interaction at a gay male private sex party in New York city. Arch Sex Behav.

[6] Goedel WC, Duncan DT (2018). Correlates of engagement in group sex events among men who have sex with men in London who use geosocial-networking smartphone applications. Int J STD AIDS.

[7] Phillips G, Magnus M, Kuo I, Rawls A, Peterson J, West-Ojo T, Jia Y, Opoku J, Greenberg AE (2014). Correlates of group sex among a community-based sample of men who have sex with men (MSM) in Washington, DC. AIDS Behav.

[8] Prestage G, Down I, Grulich A, Zablotska I (2011). Sex partying among gay men in Sydney, Melbourne and Brisbane, Australia. AIDS Behav.

[9] Kuhns LM, Hotton AL, Schneider J, Garofalo R, Fujimoto K (2017). Use of Pre-exposure Prophylaxis (PrEP) in young men who have sex with men is associated with race, sexual risk behavior and peer network size. AIDS Behav.

[10] Rice CE, Lynch CD, Norris AH, Davis JA, Fields KS, Ervin M, Turner AN (2016). Group sex and prevalent sexually transmitted infections among men who have sex with men. Arch Sex Behav.

[11] Tang W, Tang S, Qin Y, Zhang Y, Zhang W, Liu C, Tso LS, Wei C, Yang L, Huang S, Yang B, Tucker J (2016). Will gay sex-seeking mobile phone applications facilitate group sex? A cross-sectional online survey among men who have sex with men in China. PLoS One.

[12] Phillips G, Magnus M, Kuo I, Rawls A, Peterson J, Jia Y, Opoku J, Greenberg AE (2014). Use of geosocial networking (GSN) mobile phone applications to find men for sex by men who have sex with men (MSM) in Washington, DC. AIDS Behav.

[13] Friedman SR, Bolyard M, Khan M, Maslow C, Sandoval M, Mateu-Gelabert P, Krauss B, Aral SO (2008). Group sex events and HIV/STI risk in an urban network. J Acquir Immune Defic Syndr.

[14] Morris M, Goodreau S, Moody J, Holmes K, Sparling P, Stamm W, Piot P, Wasserheit J, Corey L, Cohen M (2008). Sexual networks, concurrency, STD/HIV. Sexually Transmitted Diseases. Fourth Edition.

[15] Morris M, Kretzschmar M (1995). Concurrent partnerships and transmission dynamics in networks. Soc Netw.

[16] Rich AJ, Lachowsky NJ, Cui Z, Sereda P, Lal A, Moore DM, Hogg RS, Roth EA (2016). Event-level analysis of anal sex roles and sex drug use among gay and bisexual men in Vancouver, British Columbia, Canada. Arch Sex Behav.

[17] Rice CE, Maierhofer C, Fields KS, Ervin M, Lanza ST, Turner AN (2016). Beyond anal sex: sexual practices of men who have sex with men and associations with HIV and other sexually transmitted infections. J Sex Med.

[18] Sewell J, Miltz A, Lampe FC, Cambiano V, Speakman A, Phillips AN, Stuart D, Gilson R, Asboe D, Nwokolo N, Clarke A, Collins S, Hart G, Elford J, Rodger AJ, Attitudes to Understanding of Risk of Acquisition of HIV (AURAH) Study Group (2017). Poly drug use, chemsex drug use, and associations with sexual risk behaviour in HIV-negative men who have sex with men attending sexual health clinics. Int J Drug Policy.

[19] Scheidell JD, Friedman SR, Golin C, Wohl DA, Khan MR (2017). Group sex event participation: a link to STI risk among African-American heterosexual men incarcerated in North Carolina. Sex Transm Infect.

[20] van den Boom W, Davidovich U, Heuker J, Lambers F, Prins M, Sandfort T, Stolte IG (2016). Is group sex a higher-risk setting for HIV and other sexually transmitted infections compared with dyadic sex among men who have sex with men?. Sex Transm Dis.

[21] Bérubé A (2003). The history of gay bathhouses. J Homosex.

[22] Périssé AR, Langenberg P, Hungerford L, Boulay M, Charurat M, Schechter M, Blattner W (2010). Egocentric network data provide additional information for characterizing an individual's HIV risk profile. AIDS.

[23] Delaney K (2018). Participation in group sex and associated STD risk among MSM presenting at the Seattle/King County STD clinic.

[24] Delaney K, Violette L, Ure G, Cornelius-Hudson A, Niemann L, Wesolowski L, Chavez P, Ethridge S, McMahan V, Clark H, Katz D, Stekler J (2018). Time From HIV Infection to Earliest Detection for 4 FDA-Approved Point-of-Care Tests. Proceedings of the Conference on Retroviruses and Opportunistic Infections.

[25] Sanchez TH, Sineath RC, Kahle EM, Tregear SJ, Sullivan PS (2015). The annual American men's internet survey of behaviors of men who have sex with men in the United States: protocol and key indicators report 2013. JMIR Public Health Surveill.

[26] Centers for Disease Control and Prevention.

[27] Exponential Random Graph Models (ERGMs) using statnet.

[28] Hirshfield S, Schrimshaw EW, Stall RD, Margolis AD, Downing MJ, Chiasson MA (2015). Drug use, sexual risk, and syndemic production among men who have sex with men who engage in group sexual encounters. Am J Public Health.

[29] Phillips G, Grov C, Mustanski B (2015). Engagement in group sex among geosocial networking mobile application-using men who have sex with men. Sex Health.

[30] Card KG, Lachowsky NJ, Cui Z, Shurgold S, Armstrong HL, Rich AJ, Forrest JI, Gislason M, Moore DM, Roth EA, Hogg RS (2017). An event-level analysis of the interpersonal factors associated with condomless anal sex among gay, bisexual, and other men who have sex with men (MSM) with online-met partners. AIDS Educ Prev.

[31] Pines HA, Gorbach PM, Weiss RE, Reback CJ, Landovitz RJ, Mutchler MG, Mitsuyasu RT (2016). Individual-level, partnership-level, and sexual event-level predictors of condom use during receptive anal intercourse among HIV-negative men who have sex with men in Los Angeles. AIDS Behav.

[32] Down I, Ellard J, Triffitt K, Zablotska I, Hurley M, Brown G, Bradley J, Prestage G (2017). High-risk sexual behaviours among gay and bisexual men: comparing event-level casual sex encounters among seroconverters and non-seroconverters. Sex Transm Infect.

[33] Yang C, Latkin C, Tobin K, Seal D, Koblin B, Chander G, Siconolfi D, Flores S, Spikes P (2018). An event-level analysis of condomless anal intercourse with a HIV-discordant or HIV status-unknown partner among black men who have sex with men from a multi-site study. AIDS Behav.

[34] Rendina HJ, Moody RL, Ventuneac A, Grov C, Parsons JT (2015). Aggregate and event-level associations between substance use and sexual behavior among gay and bisexual men: Comparing retrospective and prospective data. Drug Alcohol Depend.

[35] Vosburgh HW, Mansergh G, Sullivan PS, Purcell DW (2012). A review of the literature on event-level substance use and sexual risk behavior among men who have sex with men. AIDS Behav.

[36] Felsher M, Koku E (2018). Explaining HIV risk multiplexity: a social network analysis. AIDS Behav.

[37] Baral SD, Poteat T, Strömdahl S, Wirtz AL, Guadamuz TE, Beyrer C (2013). Worldwide burden of HIV in transgender women: a systematic review and meta-analysis. Lancet Infect Dis.

[38] Becasen JS, Denard CL, Mullins MM, Higa DH, Sipe TA (2019). Estimating the prevalence of HIV and sexual behaviors among the US transgender population: a systematic review and meta-analysis, 2006–2017. Am J Public Health.

[39] Poteat T, German D, Flynn C (2016). The conflation of gender and sex: gaps and opportunities in HIV data among transgender women and MSM. Glob Public Health.

[40] Younge SN, Salazar LF, Crosby RF, DiClemente RJ, Wingood GM, Rose E (2008). Condom use at last sex as a proxy for other measures of condom use: is it good enough?. Adolescence.

[41] Lachowsky NJ, Card KG, Cui Z, Sereda P, Roth EA, Hogg RS, Moore DM (2019). Agreement between gay, bisexual and other men who have sex with men's period prevalence and event-level recall of sexual behaviour: an observational respondent-driven sampling study. Sex Health.

[42] (2018). Centers for Disease Control and Prevention.

[43] King County, Washington.

