# Supplementary material for: Group Sex Events Among Cisgender Men Who Have Sex With Men: Cross-Sectional and Longitudinal Survey Study to Explore Participation and Risk-Taking Behaviors
Source: JMIR Res Protoc. 2019 Nov 27;8(11):e15426. doi: 10.2196/15426 (PMC6906620; doi:10.2196/15426)
Supplement: Multimedia Appendix 1 [file resprot_v8i11e15426_app1.pdf]

The next few questions will ask about times you had sex with more than one man in the same encounter. For these questions, a threesome involves sex with you and two other men. Group sex involves sex with you and at least 3 other men. When the question asks about the number of partners you had during a threesome or group sex, please write the total number of different partners you had across all encounters.

Don't Know

Refuse to Answer

Not Applicable

Previous Question

Next Question

Repeat the Question

In the past 3 months, how many times did you have sex with more than one man in the same encounter? (Threesome or group sex)

1	2	3	Clear
4	5	6	
7	8	9	
+/-	0	.	

Don't Know

Refuse to Answer

Not Applicable

Previous Question

Next Question

Repeat the Question

In the past 3 months, how many times did you have sex with only two other men in the same encounter (a threesome)?

1	2	3	Clear
4	5	6	
7	8	9	
+/-	0	.	

Don't Know

Refuse to Answer

Not Applicable

Previous Question

Next Question

Repeat the Question

In the past 3 months, in these 3 threesomes, with how many different men did you have anal sex?

Don't Know

Refuse to Answer

1	2	3	Clear
4	5	6	
7	8	9	
+/-	0	.	

Not Applicable

Previous Question

Next Question

Repeat the Question

In the past 3 months, of the 3 men you had anal sex with during threesomes, with how many different men did you have anal sex without a condom?

1	2	3	Clear
4	5	6	
7	8	9	
+/-	0	.	

Don't Know

Refuse to Answer

Not Applicable

Previous Question

Next Question

Repeat the Question

The next three questions are going to ask you about the HIV status (unknown, HIV positive, or HIV negative) of the 3 men with whom you had anal sex without using a condom during threesomes in the past 3 months.

Don't Know

Refuse to Answer

Not Applicable

Previous Question

Next Question

Repeat the Question

How many of these 3 men, with whom you had anal sex without using a condom during threesomes, were partners whose HIV status you did not know?

Don't Know

Refuse to Answer

1	2	3	Clear
4	5	6	
7	8	9	
+/-	0	.	

Not Applicable

Previous Question

Next Question

Repeat the Question

How many of these 3 men, with whom you had anal sex without using a condom during threesomes, were HIV positive?

1	2	3	Clear
4	5	6	
7	8	9	
+/-	0	.	

Don't Know

Refuse to Answer

Not Applicable

Previous Question

Next Question

Repeat the Question

How many of these 3 men, with whom you had anal sex without using a condom during threesomes, were HIV negative?

1	2	3	Clear	Don't Know
				Refuse to Answer
				Not Applicable
4	5	6		Previous Question
7	8	9		Next Question
+/-	0	.		Repeat the Question

In the past 3 months, how many times did you have sex with 3 or more other men in the same encounter (group sex)?

1	2	3	Clear	Don't Know
				Refuse to Answer
4	5	6		Not Applicable
				Previous Question
7	8	9		Next Question
+/-	0	.		Repeat the Question

In the past 3 months, in these 3 times you had group sex (sex with 3 or more other men), with how many different men did you have anal sex?

1	2	3	Clear	Don't Know
				Refuse to Answer
4	5	6		Not Applicable
7	8	9		Previous Question
+/-	0	.		Next Question
				Repeat the Question

In the past 3 months, of the 3 men you had anal sex with during group sex, with how many different men did you have anal sex without a condom?

1	2	3	Clear	
4	5	6		
7	8	9		
+/-	0	.		

Don't Know

Refuse to Answer

Not Applicable

Previous Question

Next Question

Repeat the Question

The next three questions are going to ask you about the HIV status (unknown, HIV positive, or HIV negative) of the 3 men with whom you had anal sex without using a condom during group sex in the past 3 months.

Don't Know

Refuse to Answer

Not Applicable

Previous Question

Next Question

Repeat the Question

How many of these 3 men, with whom you had anal sex without using a condom during group sex, were partners whose HIV status you did not know?

1	2	3	Clear	Don't Know
				Refuse to Answer
				Not Applicable
4	5	6		Previous Question
7	8	9		Next Question
+/-	0	.		Repeat the Question

How many of these 3 men, with whom you had anal sex without using a condom during group sex, were HIV positive?

1	2	3	Clear
4	5	6	
7	8	9	
+/-	0	.	

Don't Know

Refuse to Answer

Not Applicable

Previous Question

Next Question

Repeat the Question

How many of these 3 men, with whom you had anal sex without using a condom during group sex, were HIV negative?

1	2	3	Clear
4	5	6	
7	8	9	
+/-	0	.	

Don't Know

Refuse to Answer

Not Applicable

Previous Question

Next Question

Repeat the Question

In the next section we will ask you more about the 6 men you had anal sex with.

Don't Know

Refuse to Answer

Not Applicable

Previous Question

Next Question

Repeat the Question

Please enter a nickname or initials for the 3 most recent men you had **anal sex** with since *04/08/2015.* We do not want to know the names of your partners. Please choose a nickname or initials that you will remember and that will best help you identify that person.

Don't Know

Refuse to Answer

Not Applicable

Previous Question

Next Question

Repeat the Question

Please enter a nickname or initials for Partner 1:

A	B	C	D	E	F	G	Clear
H	I	J	K	L	M	N	Back
O	P	Q	R	S	T	U	Alt
V	W	X	Y	Z			

Don't Know

Refuse to Answer

Not Applicable

Previous Question

Next Question

Repeat the Question

Please enter a nickname or initials for Partner 2:

A	B	C	D	E	F	G	Clear
H	I	J	K	L	M	N	Back
O	P	Q	R	S	T	U	Alt
V	W	X	Y	Z			

Don't Know

Refuse to Answer

Not Applicable

Previous Question

Next Question

Repeat the Question

Please enter a nickname or initials for Partner 3:

A	B	C	D	E	F	G	Clear
H	I	J	K	L	M	N	Back
O	P	Q	R	S	T	U	Alt
V	W	X	Y	Z			

Don't Know

Refuse to Answer

Not Applicable

Previous Question

Next Question

Repeat the Question

Now we are going to ask you if you had sex with each of these partners during the past month, the month before that (between 05/11/2015 and 06/10/2015), and the month before that (between 04/11/2015 and 05/11/2015).

Don't Know

Refuse to Answer

Not Applicable

Previous Question

Next Question

Repeat the Question

Did you and Partner #1 have sex in the past month, that is since 06/10/2015?

NO

YES

Don't Know

Refuse to Answer

Not Applicable

Previous Question

Next Question

Repeat the Question

Did you and Partner #1 have sex 2 months ago, that is between 05/11/2015 and 06/10/2015?

NO

YES

Don't Know

Refuse to Answer

Not Applicable

Previous Question

Next Question

Repeat the Question

Did you and Partner #1 have sex 3 months ago, that is between 04/11/2015 and 05/11/2015?

Don't Know

Refuse to Answer

Not Applicable

Previous Question

Next Question

Repeat the Question

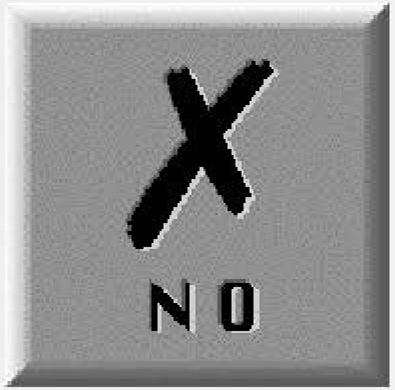

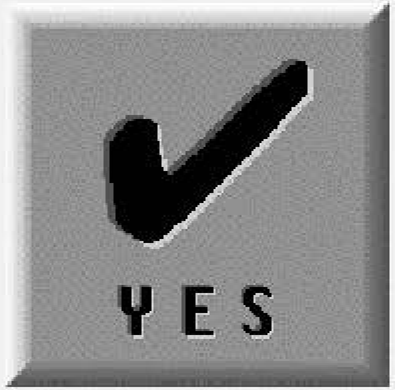

Did you and Partner #2 have sex in the past month,that is since 06/10/2015?

Don't Know

Refuse to Answer

Not Applicable

Previous Question

Next Question

Repeat the Question

NO

YES

Did you and Partner #2 have sex 2 months ago, that is between 05/11/2015 and 06/10/2015?

Don't Know

Refuse to Answer

Not Applicable

NO

YES

Previous Question

Next Question

Repeat the Question

Did you and Partner #2 have sex 3 months ago, that is between 04/11/2015 and 05/11/2015?

NO

YES

Don't Know

Refuse to Answer

Not Applicable

Previous Question

Next Question

Repeat the Question

Did you and Partner #3 have sex in the past month, that is since 06/10/2015?

NO

YES

Don't Know

Refuse to Answer

Not Applicable

Previous Question

Next Question

Repeat the Question

Did you and Partner #3 have sex 2 months ago, that is between 05/11/2015 and 06/10/2015?

Don't Know

Refuse to Answer

Not Applicable

Previous Question

Next Question

Repeat the Question

NO

YES

Did you and Partner #3 have sex 3 months ago, that is between 04/11/2015 and 05/11/2015?

NO

YES

Don't Know

Refuse to Answer

Not Applicable

Previous Question

Next Question

Repeat the Question

You indicated that you had sex with both Partner #1 and Partner #2 in the same month. Which of these statements is correct?

I last had sex with Partner #1 before I had sex with Partner #2

I last had sex with Partner #2 before I had sex with Partner #1.

I was having sex with both Partner #1 and Partner #2 during the same time period.

Don't Know

Refuse to Answer

Not Applicable

Previous Question

Next Question

Repeat the Question

You indicated that you had sex with both Partner #2 and Partner #3 in that past month.
Which of these statements is most correct?

Don't Know

Refuse to Answer

I last had sex with Partner #2 before I had sex with Partner #3

Not Applicable

I last had sex with Partner #3 before I had sex with Partner #2.

Previous Question

Next Question

I was having sex with both Partner #2 and Partner #3 during the same time period.

Repeat the Question

You indicated that you had sex with both Partner #1 and Partner #3 in the same month. Which of these statements is correct?

I last had sex with Partner #1 before I had sex with Partner #3

I last had sex with Partner #3 before I had sex with Partner #1.

I was having sex with both Partner #1 and Partner #3 during the same time period.

Don't Know

Refuse to Answer

Not Applicable

Previous Question

Next Question

Repeat the Question

Thank you for telling us the months in which you had sex with your partners. The next page will ask some questions to help us better understand what you just told us about your sexual partnerships. Click Next to continue

Don't Know

Refuse to Answer

Not Applicable

Previous Question

Next Question

Repeat the Question

Did you have sex with Partner #1 once, or more than once during the last 3 months?

Once

More than once

Don't Know

Refuse to Answer

Not Applicable

Previous Question

Next Question

Repeat the Question

Do you know the date that you first had anal sex with Partner #1?

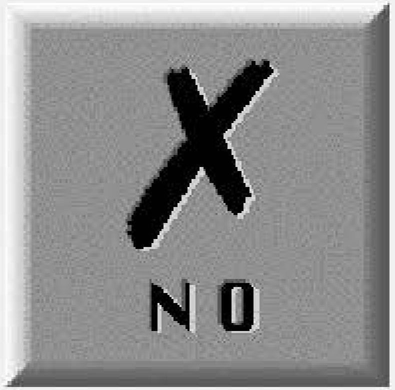

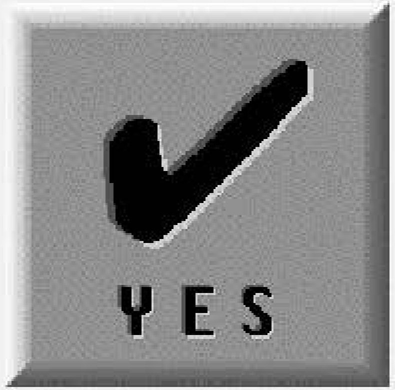

Don't Know

Refuse to Answer

Not Applicable

Previous Question

Next Question

Repeat the Question

When did you first have anal sex with Partner #1? **You may enter just the month and year**, but if you know the exact date, please enter that instead. If the first time you had sex with Partner #1 was longer than 3 months ago; we still would like to know the first time you had anal sex with him.

Year: << < [ ] > >>

Month: << < [ ] > >>

Day: << < [ ] > >>

Don't Know

Refuse to Answer

Not Applicable

Previous Question

Next Question

Repeat the Question

About how many years ago did you first have anal sex with Partner #1?

1	2	3
4	5	6
7	8	9
+/-	0	.

Clear

Years ago:

Don't Know

Refuse to Answer

Not Applicable

Previous Question

Next Question

Repeat the Question

Think back to the time when you first had sex with Partner #1 . Perhaps you had sex around a special time of the year such as your birthday, or a holiday like July 4th or Halloween. Maybe you can remember that it was warm outside or it was after a trip you took. Based on what you can recall, try to select what time you first had sex with Partner #1 :

January - March

April - June

July - September

October - December

Don't Know

Refuse to Answer

Not Applicable

Previous Question

Next Question

Repeat the Question

What is the date you last had anal sex with Partner #1? If you remember the exact date please enter it in, however if you can't please provide us with the month and year (you can leave the day section blank if needed).

Don't Know

Refuse to Answer

Year: << < > >>

Not Applicable

Month: << < > >>

Previous Question

Day: << < > >>

Next Question

Repeat the Question

Do you remember the exact date that you first had anal sex with Partner #1?

NO

YES

Don't Know

Refuse to Answer

Not Applicable

Previous Question

Next Question

Repeat the Question

When did you first have anal sex with Partner #1?

Year:	<<	<		>	>>
Month:	<<	<		>	>>
Day:	<<	<		>	>>

Don't Know

Refuse to Answer

Not Applicable

Previous Question

Next Question

Repeat the Question

Which of the following statements about Partner #1's age is most true?

Don't Know

Refuse to Answer

Not Applicable

Previous Question

Next Question

Repeat the Question

He is more than 10 years younger than I am

He is 2-10 years younger than I am

He is within a year of my age

He is 2-10 years older than I am

He is more than 10 years older than I am

Is Partner #1 Hispanic?

NO

YES

Don't Know

Refuse to Answer

Not Applicable

Previous Question

Next Question

Repeat the Question

What is Partner #1 race? Check all that apply.

Don't Know

Refuse to Answer

Not Applicable

Previous Question

Next Question

Repeat the Question

American Indian or Alaska Native

Asian

Black or African American

Native Hawaiian or Other Pacific Islander

White

Is/was Partner #1 someone that you feel or felt committed to (someone you might call your boyfriend, significant other, life partner, or husband)?

NO

YES

Don't Know

Refuse to Answer

Not Applicable

Previous Question

Next Question

Repeat the Question

If you had to further describe the type of sex partner Partner #1 is/was, which of the following would you choose? Someone who …

Is a primary sexual partner

You had sexual contact with only 1 time, but could find again if necessary

You have sex with on a regular basis, but who is not a main or primary partner

You had never met before you had had sexual contact and never plan to see again

You have had sexual contact with more than once, but not on a regular basis, and who you normally socialize with

You gave sex to for money or other goods or someone who gave you sex for money or other goods

You have had sexual contact with more than once, but not on a regular basis and who you dont socialize with

Don't Know

Refuse to Answer

Not Applicable

Previous Question

Next Question

Repeat the Question

Where did you first meet Partner #1?

Through friends	At church	On the street	Private sex party
School or work	Through a personal ad in a newspaper	Adult bookstore	Sports club or gym
Online	On a telephone chat line or dating line	Bath house	Vacation or cruise
Phone app	Bar/Club	Sex club	Social organization
Circuit party or Rave	Cruising area	Sex resort	Other

Don't Know

Refuse to Answer

Not Applicable

Previous Question

Next Question

Repeat the Question

You replied "other". Please use the laptop keyboard to type in where you first met Partner #1.

A	B	C	D	E	F	G	Clear
H	I	J	K	L	M	N	Back
O	P	Q	R	S	T	U	Alt
V	W	X	Y	Z			

Don't Know

Refuse to Answer

Not Applicable

Previous Question

Next Question

Repeat the Question

Through which online service did you first meet Partner #1?

Facebook	Manhunt	Friendster	Don't Know
CraigsList	OkCupid	MySpace	Refuse to Answer
Adam4Adam	GuySpy	Other, please specify	Not Applicable
BarebackRT	FindFred		Previous Question
			Next Question
			Repeat the Question

You replied "other". Please use the laptop keyboard to type in the online service through which you first met Partner #1.

A	B	C	D	E	F	G	Clear
H	I	J	K	L	M	N	Back
O	P	Q	R	S	T	U	Alt
V	W	X	Y	Z			

Don't Know

Refuse to Answer

Not Applicable

Previous Question

Next Question

Repeat the Question

Through which phone app did you first meet Partner #1?

Grindr	Adam4Adam	BoyAhoy
Scruff	Growlr	Other, please specify
Jackd	GuySpy	
Hornet	Skout	

Don't Know

Refuse to Answer

Not Applicable

Previous Question

Next Question

Repeat the Question

You replied "other". Please use the laptop keyboard to type in the phone app through which you first met Partner #1.

A	B	C	D	E	F	G	Clear	Refuse to Answer
H	I	J	K	L	M	N	Back	Not Applicable
O	P	Q	R	S	T	U	Alt	Previous Question
V	W	X	Y	Z				Next Question

Don't Know

Repeat the Question

Did you share your HIV status with Partner #1 before you first had sex?

Don't Know

Refuse to Answer

Not Applicable

NO

YES

Previous Question

Next Question

Repeat the Question

Did Partner #1 share his HIV status with you before you first had sex?

NO

YES

Don't Know

Refuse to Answer

Not Applicable

Previous Question

Next Question

Repeat the Question

What was Partner #1's HIV status at that time?

Don't Know

Refuse to Answer

Not Applicable

HIV-negative

Previous Question

HIV-positive

Next Question

Repeat the Question

To the best of your knowledge, what is Partner #1's HIV status today?

Don't Know

Refuse to Answer

HIV-negative

Not Applicable

Previous Question

HIV-positive

Next Question

Repeat the Question

To the best of your knowledge, is Partner #1 currently taking medicine to prevent himself from getting HIV? This might also be known as Pre-Exposure Prophylaxis or PrEP.

NO

YES

Don't Know

Refuse to Answer

Not Applicable

Previous Question

Next Question

Repeat the Question

To the best of your knowledge, is Partner #1 currently taking medicines for HIV?

Don't Know

Refuse to Answer

Not Applicable

NO

YES

Previous Question

Next Question

Repeat the Question

To the best of your knowledge, does Partner #1 have an undetectable viral load?

Don't Know

Refuse to Answer

Not Applicable

Previous Question

Next Question

Repeat the Question

NO

YES

In the past 3 months (since 04/08/2015), how many times did you and Partner #1 have anal sex?

Don't Know

Refuse to Answer

1 2 3

Clear

Not Applicable

4 5 6

Previous Question

7 8 9

Next Question

+/- 0 .

Repeat the Question

In the past 3 months (since 04/08/2015), how many times did you and Partner #1 have anal sex without using a condom or not using it the whole time?

Don't Know

Refuse to Answer

1 2 3

Clear

Not Applicable

4 5 6

Previous Question

7 8 9

Next Question

+/- 0 .

Repeat the Question

In the past 3 months (since 04/08/2015), when you and Partner #1 had anal sex without a condom were you the top (your penis in his butt), bottom (his penis in your butt) or both? Check only one.

Bottom only

Top only

Both top and bottom

Don't Know

Refuse to Answer

Not Applicable

Previous Question

Next Question

Repeat the Question

In the last 3 months (since 04/08/2015), during the time you were having sex with Partner #1, did Partner #1 have sex with anyone else?

Don't Know

Refuse to Answer

Not Applicable

Previous Question

Next Question

Repeat the Question

NO

YES

Was Partner #1 diagnosed with an STD in the last three months, since 04/08/2015?

Don't Know

Refuse to Answer

Not Applicable

Previous Question

Next Question

Repeat the Question

NO

YES

The next few questions will ask about times you and Partner #1 had sex with at least one other man together in the same encounter. For these questions, a threesome involves sex with you, Partner #1, and one other man (a total of 3 men). Group sex involves sex with you, Partner #1, and at least 2 other men (a total of 4 or more men). When the question asks about the number of partners you had during a threesome or group sex, please write the total number of different partners you had across all encounters.

Don't Know

Refuse to Answer

Not Applicable

Previous Question

Next Question

Repeat the Question

In the last 3 months (since 04/08/2015), did you and Partner #1 ever have sex with other people in the same encounter? (For an example: A threesome or group sex)

NO

YES

Don't Know

Refuse to Answer

Not Applicable

Previous Question

Next Question

Repeat the Question

In the past 3 months how many times did you and Partner #1 have sex with only one other man in the same encounter (threesome)?

Don't Know

Refuse to Answer

1	2	3	Clear
4	5	6	
7	8	9	
+/-	0	.	

Not Applicable

Previous Question

Next Question

Repeat the Question

Think of the most recent time you had a threesome with Partner #1. Did anyone have anal sex during this threesome?

NO

YES

Don't Know

Refuse to Answer

Not Applicable

Previous Question

Next Question

Repeat the Question

Did anyone have anal sex without a condom during this threesome?

Don't Know

Refuse to Answer

Not Applicable

Previous Question

Next Question

Repeat the Question

NO

YES

Who had anal sex without a condom with each other in this threesome? Mark all that apply.

I had anal sex without a condom with Partner #1

I had anal sex without a condom with the 3rd partner

Partner #1 had anal sex without a condom with the 3rd partner.

Don't Know

Refuse to Answer

Not Applicable

Previous Question

Next Question

Repeat the Question

What was the HIV status of the 3rd Partner?

HIV-Negative

HIV-Positive

Didn't know his HIV status

Don't Know

Refuse to Answer

Not Applicable

Previous Question

Next Question

Repeat the Question

In the past 3 months how many times did you and Partner #1 have sex with 2 or more other men in the same encounter (group sex)?

1	2	3	Clear
4	5	6	
7	8	9	
+/-	0	.	

Don't Know

Refuse to Answer

Not Applicable

Previous Question

Next Question

Repeat the Question

Think of the most recent time you had group sex with Partner #1 and at least two other men. How many men took part in this group sex encounter? Include Partner #1, but not yourself.

1	2	3	Clear
4	5	6	
7	8	9	
+/-	0	.	

Don't Know

Refuse to Answer

Not Applicable

Previous Question

Next Question

Repeat the Question

The next three questions are going to ask you about the HIV status (unknown, HIV positive, or HIV negative) of the 5 men involved the last time you had group sex with Partner #1.

Don't Know

Refuse to Answer

Not Applicable

Previous Question

Next Question

Repeat the Question

How many of these 5 men involved the last time you had group sex with Partner #1 were partners whose HIV status you did not know?

Don't Know

Refuse to Answer

Not Applicable

Previous Question

Next Question

Repeat the Question

1	2	3	Clear
4	5	6	
7	8	9	
+/-	0	.	

How many of these 5 men involved the last time you had group sex with Partner #1 were HIV positive?

1	2	3	Clear	Don't Know
4	5	6		Refuse to Answer
7	8	9		Not Applicable
+/-	0	.		Previous Question
				Next Question
				Repeat the Question

How many of these 5 men involved the last time you had group sex with Partner #1 were HIV negative?

1	2	3	Clear	Don't Know
				Refuse to Answer
				Not Applicable
4	5	6		Previous Question
7	8	9		Next Question
+/-	0	.		Repeat the Question

Did you and Partner #1 have anal sex without a condom during your most recent group sex encounter that included Partner #1?

Don't Know

Refuse to Answer

Not Applicable

NO

YES

Previous Question

Next Question

Repeat the Question

Did you and the 3rd partner have anal sex without a condom during your most recent group sex encounter that included Partner #1?

NO

YES

Don't Know

Refuse to Answer

Not Applicable

Previous Question

Next Question

Repeat the Question

Did you and the 4th partner have anal sex without a condom during your most recent group sex encounter that included Partner #1?

Don't Know

Refuse to Answer

Not Applicable

Previous Question

Next Question

Repeat the Question

NO

YES

Did you and the 5th partner have anal sex without a condom during your most recent group sex encounter that included Partner #1?

NO

YES

Don't Know

Refuse to Answer

Not Applicable

Previous Question

Next Question

Repeat the Question

Did Partner #1 and the 3rd partner have anal sex without a condom during your most recent group sex encounter that included Partner #1?

Don't Know

Refuse to Answer

Not Applicable

NO

YES

Previous Question

Next Question

Repeat the Question

Did Partner #1 and the 4th partner have anal sex without a condom during your most recent group sex encounter that included Partner #1?

NO

YES

Don't Know

Refuse to Answer

Not Applicable

Previous Question

Next Question

Repeat the Question

Did Partner #1 and the 5th partner have anal sex without a condom during your most recent group sex encounter that included Partner #1?

Don't Know

Refuse to Answer

Not Applicable

NO

YES

Previous Question

Next Question

Repeat the Question

Did the 3rd partner and the 4th partner have anal sex without a condom during your most recent group sex encounter that included Partner #1?

NO

YES

Don't Know

Refuse to Answer

Not Applicable

Previous Question

Next Question

Repeat the Question

Did the 3rd partner and the 5th partner have anal sex without a condom during your most recent group sex encounter that included Partner #1?

Don't Know

Refuse to Answer

Not Applicable

Previous Question

Next Question

Repeat the Question

NO

YES

Did the 4th partner and the 5th partner have anal sex without a condom during your most recent group sex encounter that included Partner #1?

NO

YES

Don't Know

Refuse to Answer

Not Applicable

Previous Question

Next Question

Repeat the Question

Did you have sex with Partner #2 once, or more than once during the last 3 months?

Once

More than once

Don't Know

Refuse to Answer

Not Applicable

Previous Question

Next Question

Repeat the Question

Do you know the date that you first had anal sex with Partner #2?

Don't Know

Refuse to Answer

Not Applicable

NO

YES

Previous Question

Next Question

Repeat the Question

When did you first have anal sex with Partner #2? **You may enter just the month and year**, but if you know the exact date, please enter that instead. If the first time you had sex with Partner #2 was longer than 3 months ago; we still would like to know the first time you had anal sex with him.

Year: << < [ ] > >>

Month: << < [ ] > >>

Day: << < [ ] > >>

Don't Know

Refuse to Answer

Not Applicable

Previous Question

Next Question

Repeat the Question

About how many years ago did you first have anal sex with Partner #2?

1	2	3
4	5	6
7	8	9
+/-	0	.

Clear

Years ago:

Don't Know

Refuse to Answer

Not Applicable

Previous Question

Next Question

Repeat the Question

Think back to the time when you first had sex with Partner #2 . Perhaps you had sex around a special time of the year such as your birthday, or a holiday like July 4th or Halloween. Maybe you can remember that it was warm outside or it was after a trip you took. Based on what you can recall, try to select what time you first had sex with Partner #2:

January - March

April - June

July - September

October - December

Don't Know

Refuse to Answer

Not Applicable

Previous Question

Next Question

Repeat the Question

What is the date you last had anal sex last had sex with Partner #2? If you remember the exact date please enter it in, however if you can't please provide us with the month and year (you can leave the day section blank if needed).

Don't Know

Refuse to Answer

Not Applicable

Previous Question

Next Question

Repeat the Question

Year: << < > >>

Month: << < > >>

Day: << < > >>

Do you remember the exact date that you first had anal sex with Partner #2?

NO

YES

Don't Know

Refuse to Answer

Not Applicable

Previous Question

Next Question

Repeat the Question

When did you first have anal sex with Partner #2?

Year: << < > >>

Month: << < > >>

Day: << < > >>

Don't Know

Refuse to Answer

Not Applicable

Previous Question

Next Question

Repeat the Question

Which of the following statements about Partner #2's age is most true?

He is more than 10 years younger than I am

He is 2-10 years younger than I am

He is within a year of my age

He is 2-10 years older than I am

He is more than 10 years older than I am

Don't Know

Refuse to Answer

Not Applicable

Previous Question

Next Question

Repeat the Question

Is Partner #2 Hispanic?

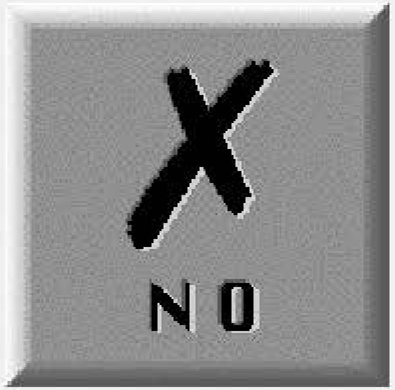

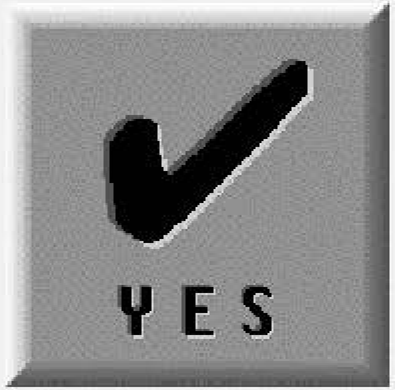

Don't Know

Refuse to Answer

Not Applicable

Previous Question

Next Question

Repeat the Question

What is Partner #2 race? Check all that apply.

American Indian or Alaska Native

Asian

Black or African American

Native Hawaiian or Other Pacific Islander

White

Don't Know

Refuse to Answer

Not Applicable

Previous Question

Next Question

Repeat the Question

Is/was Partner #2 someone that you feel or felt committed to (someone you might call your boyfriend, significant other, life partner, or husband)?

Don't Know

Refuse to Answer

Not Applicable

Previous Question

Next Question

Repeat the Question

NO

YES

If you had to further describe the type of sex partner Partner #2 is/was, which of the following would you choose? Someone who ...

Don't Know

Refuse to Answer

Is a primary sexual partner

You had sexual contact with only 1 time, but could find again if necessary

Not Applicable

You have sex with on a regular basis, but who is not a main or primary partner

You had never met before you had had sexual contact and never plan to see again

Previous Question

You have had sexual contact with more than once, but not on a regular basis, and who you normally socialize with

You gave sex to for money or other goods or someone who gave you sex for money or other goods

Next Question

You have had sexual contact with more than once, but not on a regular basis and who you dont socialize with

Repeat the Question

Where did you first meet Partner #2?

Through friends	At church	On the street	Private sex party
School or work	Through a personal ad in a newspaper	Adult bookstore	Sports club or gym
Online	On a telephone chat line or dating line	Bath house	Vacation or cruise
Phone app	Bar/Club	Sex club	Social organization
Circuit party or Rave	Cruising area	Sex resort	Other

Don't Know

Refuse to Answer

Not Applicable

Previous Question

Next Question

Repeat the Question

You replied "other". Please use the laptop keyboard to type in where you first met Partner #2.

A	B	C	D	E	F	G	Clear
H	I	J	K	L	M	N	Back
O	P	Q	R	S	T	U	Alt
V	W	X	Y	Z			

Don't Know

Refuse to Answer

Not Applicable

Previous Question

Next Question

Repeat the Question

Through which online service did you first meet Partner #2?

Facebook	Manhunt	Friendster
CraigsList	OkCupid	MySpace
Adam4Adam	GuySpy	Other, please specify
BarebackRT	FindFred	

Don't Know

Refuse to Answer

Not Applicable

Previous Question

Next Question

Repeat the Question

You replied "other". Please use the laptop keyboard to type in the online service through which you first met Partner #2.

A	B	C	D	E	F	G	Clear
H	I	J	K	L	M	N	Back
O	P	Q	R	S	T	U	Alt
V	W	X	Y	Z			

Don't Know

Refuse to Answer

Not Applicable

Previous Question

Next Question

Repeat the Question

Through which phone app did you first meet Partner #2?

Grindr	Adam4Adam	BoyAhoy
Scruff	Growlr	Other, please specify
Jackd	GuySpy	
Hornet	Skout	

Don't Know

Refuse to Answer

Not Applicable

Previous Question

Next Question

Repeat the Question

You replied "other". Please use the laptop keyboard to type in the phone app through which you first met Partner #2.

A	B	C	D	E	F	G	Clear
H	I	J	K	L	M	N	Back
O	P	Q	R	S	T	U	Alt
V	W	X	Y	Z			

Don't Know

Refuse to Answer

Not Applicable

Previous Question

Next Question

Repeat the Question

Did you share your HIV status with Partner #2 before you first had sex?

Don't Know

Refuse to Answer

Not Applicable

NO

YES

Previous Question

Next Question

Repeat the Question

Did Partner #2 share his HIV status with you before you first had sex?

Don't Know

Refuse to Answer

Not Applicable

Previous Question

Next Question

Repeat the Question

NO

YES

What was Partner #2's HIV status at that time?

HIV-negative

HIV-positive

Don't Know

Refuse to Answer

Not Applicable

Previous Question

Next Question

Repeat the Question

To the best of your knowledge, what is Partner #2's HIV status today?

HIV-negative

HIV-positive

Don't Know

Refuse to Answer

Not Applicable

Previous Question

Next Question

Repeat the Question

To the best of your knowledge, is Partner #2 currently taking medicine to prevent himself from getting HIV? This might also be known as Pre-Exposure Prophylaxis or PrEP.

Don't Know

Refuse to Answer

Not Applicable

Previous Question

Next Question

Repeat the Question

NO

YES

To the best of your knowledge, is Partner #2 currently taking medicines for HIV?

Don't Know

Refuse to Answer

Not Applicable

NO

YES

Previous Question

Next Question

Repeat the Question

To the best of your knowledge, does Partner #2 have an undetectable viral load?

NO

YES

Don't Know

Refuse to Answer

Not Applicable

Previous Question

Next Question

Repeat the Question

In the past 3 months (since 04/08/2015), how many times did you and Partner #2 have anal sex?

Don't Know

Refuse to Answer

1 2 3 Clear

Not Applicable

4 5 6

Previous Question

7 8 9

Next Question

+/- 0 .

Repeat the Question

In the past 3 months (since 04/08/2015), how many times did you and Partner #2 have anal sex without using a condom or not using it the whole time?

1	2	3	Clear	Don't Know
				Refuse to Answer
4	5	6		Not Applicable
				Previous Question
7	8	9		Next Question
+/-	0	.		Repeat the Question

In the past 3 months (since 04/08/2015), when you and Partner #2 had anal sex without a condom were you the top (your penis in his butt), bottom (his penis in your butt) or both? Check only one.

Bottom only

Top only

Both top and bottom

Don't Know

Refuse to Answer

Not Applicable

Previous Question

Next Question

Repeat the Question

In the last 3 months (since 04/08/2015), during the time you were having sex with Partner #2, did Partner #2 have sex with anyone else?

NO

YES

Don't Know

Refuse to Answer

Not Applicable

Previous Question

Next Question

Repeat the Question

Was Partner #2 diagnosed with an STD in the last three months, since 04/08/2015?

NO

YES

Don't Know

Refuse to Answer

Not Applicable

Previous Question

Next Question

Repeat the Question

The next few questions will ask about times you and Partner #2 had sex with at least one other man together in the same encounter. For these questions, a threesome involves sex with you, Partner #2, and one other man (a total of 3 men). Group sex involves sex with you, Partner #2, and at least 2 other men (a total of 4 or more men). When the question asks about the number of partners you had during a threesome or group sex, please write the total number of different partners you had across all encounters.

Don't Know

Refuse to Answer

Not Applicable

Previous Question

Next Question

Repeat the Question

In the last 3 months (since 04/08/2015), did you and Partner #2 ever have sex with other people in the same encounter? (For an example: A threesome or group sex)

Don't Know

Refuse to Answer

Not Applicable

NO

YES

Previous Question

Next Question

Repeat the Question

In the past 3 months how many times did you and Partner #2 have sex with only one other man in the same encounter (threesome)?

1	2	3	Clear
4	5	6	
7	8	9	
+/-	0	.	

Don't Know

Refuse to Answer

Not Applicable

Previous Question

Next Question

Repeat the Question

Think of the most recent time you had a threesome with Partner #2. Did anyone have anal sex during this threesome?

NO

YES

Don't Know

Refuse to Answer

Not Applicable

Previous Question

Next Question

Repeat the Question

Did anyone have anal sex without a condom during this threesome?

Don't Know

Refuse to Answer

Not Applicable

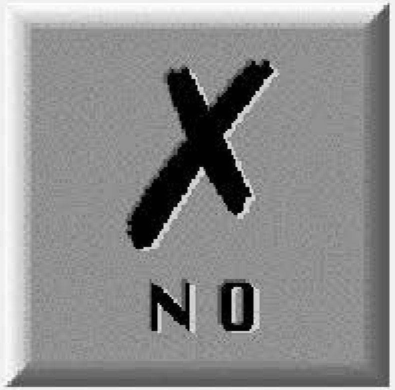

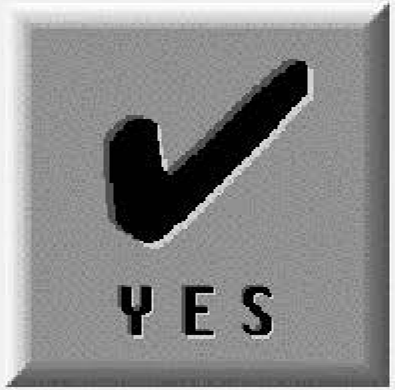

Previous Question

Next Question

Repeat the Question

Who had anal sex without a condom with each other in this threesome? Mark all that apply.

I had anal sex without a condom with Partner #2

I had anal sex without a condom with the 3rd partner

Partner #2 had anal sex without a condom with the 3rd partner.

Don't Know

Refuse to Answer

Not Applicable

Previous Question

Next Question

Repeat the Question

What was the HIV status of the 3rd Partner?

HIV-Negative

HIV-Positive

Didn't know his HIV status

Don't Know

Refuse to Answer

Not Applicable

Previous Question

Next Question

Repeat the Question

In the past 3 months how many times did you and Partner #2 have sex with 2 or more other men in the same encounter (group sex)?

1	2	3	Clear
4	5	6	
7	8	9	
+/-	0	.	

Don't Know

Refuse to Answer

Not Applicable

Previous Question

Next Question

Repeat the Question

Think of the most recent time you had group sex with Partner #2 and at least two other men. How many men took part in this group sex encounter? Include Partner #2, but not yourself.

1	2	3	Clear	
4	5	6		
7	8	9		
+/-	0	.		

Don't Know

Refuse to Answer

Not Applicable

Previous Question

Next Question

Repeat the Question

The next three questions are going to ask you about the HIV status (unknown, HIV positive, or HIV negative) of the 5 men involved the last time you had group sex with Partner #2.

Don't Know

Refuse to Answer

Not Applicable

Previous Question

Next Question

Repeat the Question

How many of these 5 men involved the last time you had group sex with Partner #2 were partners whose HIV status you did not know?

Don't Know

Refuse to Answer

1	2	3	Clear
4	5	6	
7	8	9	
+/-	0	.	

Not Applicable

Previous Question

Next Question

Repeat the Question

How many of these 5 men involved the last time you had group sex with Partner #2 were HIV positive?

1	2	3	Clear	Don't Know
4	5	6		Refuse to Answer
7	8	9		Not Applicable
+/-	0	.		Previous Question
				Next Question
				Repeat the Question

How many of these 5 men involved the last time you had group sex with Partner #2 were HIV negative?

1	2	3	Clear
4	5	6	
7	8	9	
+/-	0	.	

Don't Know

Refuse to Answer

Not Applicable

Previous Question

Next Question

Repeat the Question

Did you and Partner #2 have anal sex without a condom during your most recent group sex encounter that included Partner #2?

NO

YES

Don't Know

Refuse to Answer

Not Applicable

Previous Question

Next Question

Repeat the Question

Did you and the 3rd partner have anal sex without a condom during your most recent group sex encounter that included Partner #2?

NO

YES

Don't Know

Refuse to Answer

Not Applicable

Previous Question

Next Question

Repeat the Question

Did you and the 4th partner have anal sex without a condom during your most recent group sex encounter that included Partner #2?

NO

YES

Don't Know

Refuse to Answer

Not Applicable

Previous Question

Next Question

Repeat the Question

Did you and the 5th partner have anal sex without a condom during your most recent group sex encounter that included Partner #2?

Don't Know

Refuse to Answer

Not Applicable

Previous Question

Next Question

Repeat the Question

NO

YES

Did Partner #2 and the 3rd partner have anal sex without a condom during your most recent group sex encounter that included Partner #2?

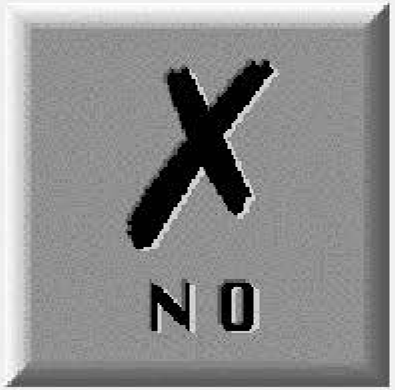

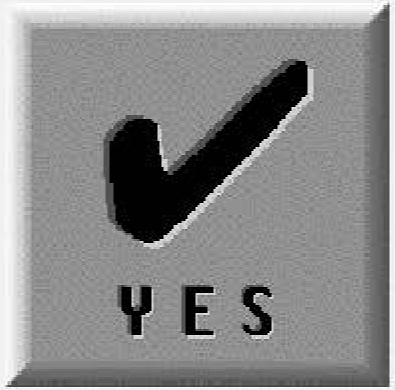

Don't Know

Refuse to Answer

Not Applicable

Previous Question

Next Question

Repeat the Question

Did Partner #2 and the 4th partner have anal sex without a condom during your most recent group sex encounter that included Partner #2?

NO

YES

Don't Know

Refuse to Answer

Not Applicable

Previous Question

Next Question

Repeat the Question

Did Partner #2 and the 5th partner have anal sex without a condom during your most recent group sex encounter that included Partner #2?

Don't Know

Refuse to Answer

Not Applicable

NO

YES

Previous Question

Next Question

Repeat the Question

Did the 3rd partner and the 4th partner have anal sex without a condom during your most recent group sex encounter that included Partner #2?

Don't Know

Refuse to Answer

Not Applicable

Previous Question

Next Question

Repeat the Question

NO

YES

Did the 3rd partner and the 5th partner have anal sex without a condom during your most recent group sex encounter that included Partner #2?

Don't Know

Refuse to Answer

Not Applicable

Previous Question

Next Question

Repeat the Question

NO

YES

Did the 4th partner and the 5th partner have anal sex without a condom during your most recent group sex encounter that included Partner #2?

Don't Know

Refuse to Answer

Not Applicable

Previous Question

Next Question

Repeat the Question

NO

YES

Did you have sex with Partner #3 once, or more than once during the last 3 months?

Once

More than once

Don't Know

Refuse to Answer

Not Applicable

Previous Question

Next Question

Repeat the Question

Do you know the date that you first had anal sex with Partner #3?

Don't Know

Refuse to Answer

Not Applicable

Previous Question

Next Question

Repeat the Question

NO

YES

When did you first have anal sex with Partner #3? **You may enter just the month and year**, but if you know the exact date, please enter that instead. If the first time you had sex with Partner #3 was longer than 3 months ago; we still would like to know the first time you had anal sex with him.

Year:	<<	<		>	>>	
Month:	<<	<		>	>>	
Day:	<<	<		>	>>	

Don't Know

Refuse to Answer

Not Applicable

Previous Question

Next Question

Repeat the Question

About how many years ago did you first have anal sex with Partner #3?

1	2	3
4	5	6
7	8	9
+/-	0	.

Clear

Years ago:

Don't Know

Refuse to Answer

Not Applicable

Previous Question

Next Question

Repeat the Question

Think back to the time when you first had sex with Partner #3 . Perhaps you had sex around a special time of the year such as your birthday, or a holiday like July 4th or Halloween. Maybe you can remember that it was warm outside or it was after a trip you took. Based on what you can recall, try to select what time you first had sex with Partner #3 :

January - March

April - June

July - September

October - December

Don't Know

Refuse to Answer

Not Applicable

Previous Question

Next Question

Repeat the Question

What is the date you last had anal sex with Partner #3? If you remember the exact date please enter it in, however if you can't please provide us with the month and year (you can leave the day section blank if needed).

Year:	<<	<		>	>>
Month:	<<	<		>	>>
Day:	<<	<		>	>>

Don't Know

Refuse to Answer

Not Applicable

Previous Question

Next Question

Repeat the Question

Do you remember the exact date that you first had anal sex with Partner #3?

NO

YES

Don't Know

Refuse to Answer

Not Applicable

Previous Question

Next Question

Repeat the Question

When did you first have anal sex with Partner #3?

Year:	<<	<		>	>>
Month:	<<	<		>	>>
Day:	<<	<		>	>>

Don't Know

Refuse to Answer

Not Applicable

Previous Question

Next Question

Repeat the Question

Which of the following statements about Partner #3's age is most true?

He is more than 10 years younger than I am

He is 2-10 years younger than I am

He is within a year of my age

He is 2-10 years older than I am

He is more than 10 years older than I am

Don't Know

Refuse to Answer

Not Applicable

Previous Question

Next Question

Repeat the Question

Is Partner #3 Hispanic?

NO

YES

Don't Know

Refuse to Answer

Not Applicable

Previous Question

Next Question

Repeat the Question

What is Partner #3 race? Check all that apply.

Don't Know

Refuse to Answer

Not Applicable

Previous Question

Next Question

Repeat the Question

American Indian or Alaska Native

Asian

Black or African American

Native Hawaiian or Other Pacific Islander

White

Is/was Partner #3 someone that you feel or felt committed to (someone you might call your boyfriend, significant other, life partner, or husband)?

NO

YES

Don't Know

Refuse to Answer

Not Applicable

Previous Question

Next Question

Repeat the Question

If you had to further describe the type of sex partner Partner #3 is/was, which of the following would you choose? Someone who …

- Is a primary sexual partner
- You had sexual contact with only 1 time, but could find again if necessary
- You have sex with on a regular basis, but who is not a main or primary partner
- You had never met before you had had sexual contact and never plan to see again
- You have had sexual contact with more than once, but not on a regular basis, and who you normally socialize with
- You gave sex to for money or other goods or someone who gave you sex for money or other goods
- You have had sexual contact with more than once, but not on a regular basis and who you dont socialize with

Don't Know

Refuse to Answer

Not Applicable

Previous Question

Next Question

Repeat the Question

Where did you first meet Partner #3?

Through friends	At church	On the street	Private sex party
School or work	Through a personal ad in a newspaper	Adult bookstore	Sports club or gym
Online	On a telephone chat line or dating line	Bath house	Vacation or cruise
Phone app	Bar/Club	Sex club	Social organization
Circuit party or Rave	Cruising area	Sex resort	Other

Don't Know

Refuse to Answer

Not Applicable

Previous Question

Next Question

Repeat the Question

You replied "other". Please use the laptop keyboard to type in where you first met Partner #3.

A	B	C	D	E	F	G	Clear
H	I	J	K	L	M	N	Back
O	P	Q	R	S	T	U	Alt
V	W	X	Y	Z			

Don't Know

Refuse to Answer

Not Applicable

Previous Question

Next Question

Repeat the Question

Through which online service did you first meet Partner #3?

Facebook	Manhunt	Friendster
CraigsList	OkCupid	MySpace
Adam4Adam	GuySpy	Other, please specify
BarebackRT	FindFred	

Don't Know

Refuse to Answer

Not Applicable

Previous Question

Next Question

Repeat the Question

You replied "other". Please use the laptop keyboard to type in the online service through which you first met Partner #3.

A	B	C	D	E	F	G	Clear
H	I	J	K	L	M	N	Back
O	P	Q	R	S	T	U	Alt
V	W	X	Y	Z			

Don't Know

Refuse to Answer

Not Applicable

Previous Question

Next Question

Repeat the Question

Through which phone app did you first meet Partner #3?

Grindr	Adam4Adam	BoyAhoy
Scruff	Growlr	Other, please specify
Jackd	GuySpy	
Hornet	Skout	

Don't Know

Refuse to Answer

Not Applicable

Previous Question

Next Question

Repeat the Question

You replied "other". Please use the laptop keyboard to type in the phone app through which you first met Partner #3.

A	B	C	D	E	F	G	Clear
H	I	J	K	L	M	N	Back
O	P	Q	R	S	T	U	Alt
V	W	X	Y	Z			

Don't Know

Refuse to Answer

Not Applicable

Previous Question

Next Question

Repeat the Question

Did you share your HIV status with Partner #3 before you first had sex?

Don't Know

Refuse to Answer

Not Applicable

NO

YES

Previous Question

Next Question

Repeat the Question

Did Partner #3 share his HIV status with you before you first had sex?

Don't Know

Refuse to Answer

Not Applicable

Previous Question

Next Question

Repeat the Question

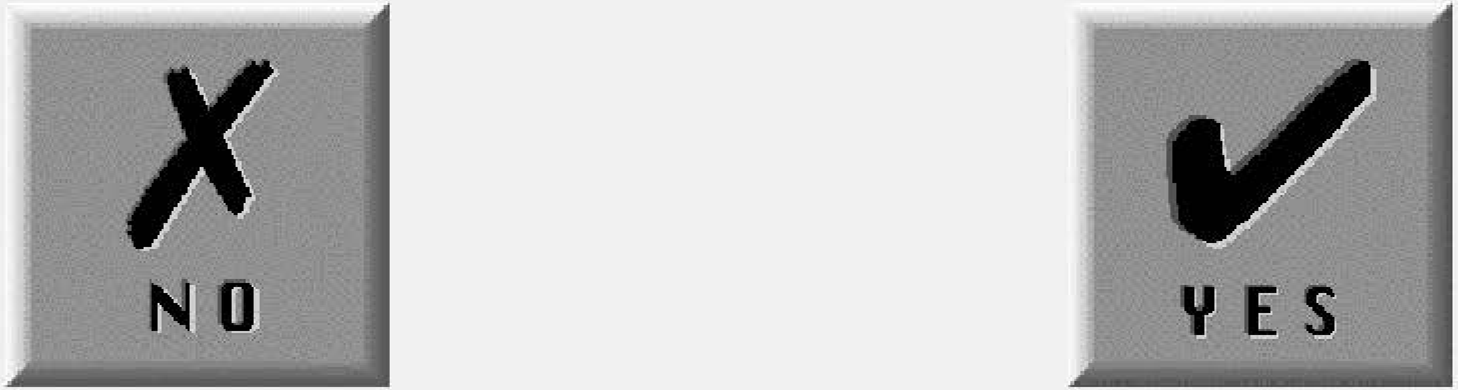

What was Partner #3's HIV status at that time?

HIV-negative

HIV-positive

Don't Know

Refuse to Answer

Not Applicable

Previous Question

Next Question

Repeat the Question

To the best of your knowledge, what is Partner #3's HIV status today?

Don't Know

Refuse to Answer

Not Applicable

HIV-negative

Previous Question

HIV-positive

Next Question

Repeat the Question

To the best of your knowledge, is Partner #3 currently taking medicine to prevent himself from getting HIV? This might also be known as Pre-Exposure Prophylaxis or PrEP.

Don't Know

Refuse to Answer

Not Applicable

Previous Question

Next Question

Repeat the Question

NO

YES

To the best of your knowledge, is Partner #3 currently taking medicines for HIV?

NO

YES

Don't Know

Refuse to Answer

Not Applicable

Previous Question

Next Question

Repeat the Question

To the best of your knowledge, does Partner #3 have an undetectable viral load?

NO

YES

Don't Know

Refuse to Answer

Not Applicable

Previous Question

Next Question

Repeat the Question

In the past 3 months (since 04/08/2015), how many times did you and Partner #3 have anal sex?

1	2	3	Clear	Don't Know
				Refuse to Answer
4	5	6		Not Applicable
				Previous Question
7	8	9		Next Question
+/-	0	.		Repeat the Question

In the past 3 months (since 04/08/2015), how many times did you and Partner #3 have anal sex without using a condom or not using it the whole time?

Don't Know

Refuse to Answer

1	2	3	Clear
4	5	6	
7	8	9	
+/-	0	.	

Not Applicable

Previous Question

Next Question

Repeat the Question

In the past 3 months (since 04/08/2015), when you and Partner #3 had anal sex without a condom were you the top (your penis in his butt), bottom (his penis in your butt) or both? Check only one.

Bottom only

Top only

Both top and bottom

Don't Know

Refuse to Answer

Not Applicable

Previous Question

Next Question

Repeat the Question

In the last 3 months (since 04/08/2015), during the time you were having sex with Partner #3, did Partner #3 have sex with anyone else?

Don't Know

Refuse to Answer

Not Applicable

Previous Question

Next Question

Repeat the Question

NO

YES

Was Partner #3 diagnosed with an STD in the last three months, since 04/08/2015?

Don't Know

Refuse to Answer

Not Applicable

NO

YES

Previous Question

Next Question

Repeat the Question

The next few questions will ask about times you and Partner #3 had sex with at least one other man together in the same encounter. For these questions, a threesome involves sex with you, Partner #3, and one other man (a total of 3 men). Group sex involves sex with you, Partner #3, and at least 2 other men (a total of 4 or more men). When the question asks about the number of partners you had during a threesome or group sex, please write the total number of different partners you had across all encounters.

Don't Know

Refuse to Answer

Not Applicable

Previous Question

Next Question

Repeat the Question

In the last 3 months (since 04/08/2015), did you and Partner #3 ever have sex with other people in the same encounter? (For an example: A threesome or group sex)

Don't Know

Refuse to Answer

Not Applicable

Previous Question

Next Question

Repeat the Question

NO

YES

In the past 3 months how many times did you and Partner #3 have sex with only one other man in the same encounter (threesome)?

1	2	3	Clear
4	5	6	
7	8	9	
+/-	0	.	

Don't Know

Refuse to Answer

Not Applicable

Previous Question

Next Question

Repeat the Question

Think of the most recent time you had a threesome with Partner #3. Did anyone have anal sex during this threesome?

NO

YES

Don't Know

Refuse to Answer

Not Applicable

Previous Question

Next Question

Repeat the Question

Did anyone have anal sex without a condom during this threesome?

Don't Know

Refuse to Answer

Not Applicable

Previous Question

Next Question

Repeat the Question

NO

YES

Who had anal sex without a condom with each other in this threesome? Mark all that apply.

I had anal sex without a condom with Partner #3

I had anal sex without a condom with the 3rd partner

Partner #3 had anal sex without a condom with the 3rd partner.

Don't Know

Refuse to Answer

Not Applicable

Previous Question

Next Question

Repeat the Question

What was the HIV status of the 3rd Partner?

HIV-Negative

HIV-Positive

Didn't know his HIV status

Don't Know

Refuse to Answer

Not Applicable

Previous Question

Next Question

Repeat the Question

In the past 3 months how many times did you and Partner #3 have sex with 2 or more other men in the same encounter (group sex)?

1	2	3	Clear
4	5	6	
7	8	9	
+/-	0	.	

Don't Know

Refuse to Answer

Not Applicable

Previous Question

Next Question

Repeat the Question

Think of the most recent time you had group sex with Partner #3 and at least two other men. How many men took part in this group sex encounter? Include Partner #3, but not yourself.

1	2	3	Clear
4	5	6	
7	8	9	
+/-	0	.	

Don't Know

Refuse to Answer

Not Applicable

Previous Question

Next Question

Repeat the Question

The next three questions are going to ask you about the HIV status (unknown, HIV positive, or HIV negative) of the 5 men involved the last time you had group sex with Partner #3.

Don't Know

Refuse to Answer

Not Applicable

Previous Question

Next Question

Repeat the Question

How many of these 5 men involved the last time you had group sex with Partner #3 were partners whose HIV status you did not know?

1	2	3	Clear
4	5	6	
7	8	9	
+/-	0	.	

Don't Know

Refuse to Answer

Not Applicable

Previous Question

Next Question

Repeat the Question

How many of these 5 men involved the last time you had group sex with Partner #3 were HIV positive?

1	2	3	Clear	Don't Know
				Refuse to Answer
4	5	6		Not Applicable
7	8	9		Previous Question
+/-	0	.		Next Question
				Repeat the Question

How many of these 5 men involved the last time you had group sex with Partner #3 were HIV negative?

1	2	3	Clear
4	5	6	
7	8	9	
+/-	0	.	

Don't Know

Refuse to Answer

Not Applicable

Previous Question

Next Question

Repeat the Question

Did you and Partner #3 have anal sex without a condom during your most recent group sex encounter that included Partner #3?

NO

YES

Don't Know

Refuse to Answer

Not Applicable

Previous Question

Next Question

Repeat the Question

Did you and the 3rd partner have anal sex without a condom during your most recent group sex encounter that included Partner #3?

Don't Know

Refuse to Answer

Not Applicable

Previous Question

Next Question

Repeat the Question

NO

YES

Did you and the 4th partner have anal sex without a condom during your most recent group sex encounter that included Partner #3?

NO

YES

Don't Know

Refuse to Answer

Not Applicable

Previous Question

Next Question

Repeat the Question

Did you and the 5th partner have anal sex without a condom during your most recent group sex encounter that included Partner #3?

Don't Know

Refuse to Answer

Not Applicable

NO

YES

Previous Question

Next Question

Repeat the Question

Did Partner #3 and the 3rd partner have anal sex without a condom during your most recent group sex encounter that included Partner #3?

NO

YES

Don't Know

Refuse to Answer

Not Applicable

Previous Question

Next Question

Repeat the Question

Did Partner #3 and the 4th partner have anal sex without a condom during your most recent group sex encounter that included Partner #3?

NO

YES

Don't Know

Refuse to Answer

Not Applicable

Previous Question

Next Question

Repeat the Question

Did Partner #3 and the 5th partner have anal sex without a condom during your most recent group sex encounter that included Partner #3?

NO

YES

Don't Know

Refuse to Answer

Not Applicable

Previous Question

Next Question

Repeat the Question

Did the 3rd partner and the 4th partner have anal sex without a condom during your most recent group sex encounter that included Partner #3?

Don't Know

Refuse to Answer

Not Applicable

NO

YES

Previous Question

Next Question

Repeat the Question

Did the 3rd partner and the 5th partner have anal sex without a condom during your most recent group sex encounter that included Partner #3?

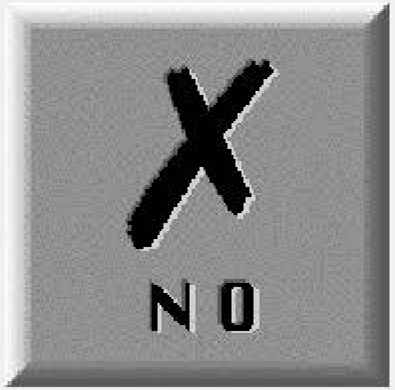

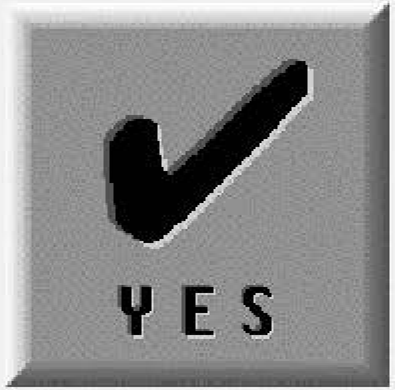

Don't Know

Refuse to Answer

Not Applicable

Previous Question

Next Question

Repeat the Question

Did the 4th partner and the 5th partner have anal sex without a condom during your most recent group sex encounter that included Partner #3?

NO

YES

Don't Know

Refuse to Answer

Not Applicable

Previous Question

Next Question

Repeat the Question

Do you know if Partner #1 had sex with Partner #2 in the last 3 months, or think that they probably have?

NO

YES

Don't Know

Refuse to Answer

Not Applicable

Previous Question

Next Question

Repeat the Question

Do you know if Partner #1 had sex with Partner #3 in the last 3 months, or think that they probably have?

NO

YES

Don't Know

Refuse to Answer

Not Applicable

Previous Question

Next Question

Repeat the Question

Do you know if Partner #2 had sex with Partner #3 in the last 3 months, or think that they probably have?

NO

YES

Don't Know

Refuse to Answer

Not Applicable

Previous Question

Next Question

Repeat the Question
