# Supplementary material for: Group Sex Events Among Cisgender Men Who Have Sex With Men: Cross-Sectional and Longitudinal Survey Study to Explore Participation and Risk-Taking Behaviors
Source: JMIR Res Protoc. 2019 Nov 27;8(11):e15426. doi: 10.2196/15426 (PMC6906620; doi:10.2196/15426)
Supplement: Multimedia Appendix 2 [file resprot_v8i11e15426_app2.pdf]

Las siguientes preguntas indagarán sobre las veces que tuvo sexo con más de un hombre en el mismo encuentro. Para estas preguntas, un trío implica sexo con usted y con otros dos hombres. El sexo grupal implica el sexo con usted y al menos otros 3 hombres. Cuando la pregunta indague sobre la cantidad de parejas que tuvo durante un trío o sexo grupal, escriba el número total de parejas diferentes que tuvo en todos los encuentros.

No Sabe

Rehusó Contestar

No es aplicable

Pregunta Previa

Siguiente Pregunta

Repita la pregunta

¿Cuántas veces tuvo sexo con más de un hombre en el mismo encuentro en los últimos 3 meses? (Trío o sexo grupal)

No Sabe

Rehusó Contestar

1 2 3

Borre

No es aplicable

4 5 6

Pregunta Previa

7 8 9

Siguiente Pregunta

+/- 0 .

Repita la pregunta

¿Cuántas veces tuvo sexo con solo otros dos hombres en el mismo encuentro (un trío) en los últimos 3 meses?

1	2	3	Borre
4	5	6	
7	8	9	
+/-	0	.	

No Sabe

Rehusó Contestar

No es aplicable

Pregunta Previa

Siguiente Pregunta

Repita la pregunta

En estos 3 tríos, ¿con cuántos hombres diferentes tuvo sexo anal en los últimos 3 meses?

No Sabe

Rehusó Contestar

1	2	3	Borre
4	5	6	
7	8	9	
+/-	0	.	

No es aplicable

Pregunta Previa

Siguiente Pregunta

Repita la pregunta

De los 3 hombres con quienes tuvo sexo anal durante los tríos, ¿con cuántos hombres diferentes tuvo sexo anal sin condón en los últimos 3 meses?

No Sabe

Rehusó Contestar

1	2	3	Borre
4	5	6	
7	8	9	
+/-	0	.	

No es aplicable

Pregunta Previa

Siguiente Pregunta

Repita la pregunta

Las siguientes tres preguntas indagarán sobre el estado de VIH (desconocido, VIH positivo o VIH negativo) de los 3 hombres con quienes tuvo sexo anal sin usar condón durante tríos en los últimos 3 meses. El número total de parejas que usted ingrese en las proximas tres preguntas se debe sumar a 3. Si ninguna de estas 3 parejas se puede ser descrita con una de las categorias del estatus de VIH, por favor ingresa 0 para esa categoría.

No Sabe

Rehusó Contestar

No es aplicable

Pregunta Previa

Siguiente Pregunta

Repita la pregunta

¿Cuántos de estos 3 hombres con quienes tuvo sexo anal sin usar condón durante los tríos fueron parejas cuyo estado de VIH desconocía?

1	2	3	Borre	No Sabe
4	5	6		Rehusó Contestar
7	8	9		No es aplicable
+/-	0	.		Pregunta Previa
				Siguiente Pregunta
				Repita la pregunta

¿Cuántos de estos 3 hombres con quienes tuvo sexo anal sin usar condón durante los tríos fueron VIH positivo?

No Sabe

Rehusó Contestar

1	2	3	Borre
4	5	6	
7	8	9	
+/-	0	.	

No es aplicable

Pregunta Previa

Siguiente Pregunta

Repita la pregunta

¿Cuántos de estos 3 hombres con quienes tuvo sexo anal sin usar condón durante los tríos fueron VIH negativo?

1	2	3	Borre	No Sabe
				Rehusó Contestar
4	5	6		No es aplicable
				Pregunta Previa
7	8	9		Siguiente Pregunta
+/-	0	.		Repita la pregunta

¿Cuántas veces tuvo sexo con 3 o más hombres en el mismo encuentro (sexo grupal) en los últimos 3 meses?

No Sabe

Rehusó Contestar

1	2	3	Borre
4	5	6	
7	8	9	
+/-	0	.	

No es aplicable

Pregunta Previa

Siguiente Pregunta

Repita la pregunta

En los últimos 3 meses, en estas 1 veces que tuvo sexo grupal (sexo con 3 o más hombres), ¿con cuántos hombres diferentes tuvo sexo anal?

No Sabe

Rehusó Contestar

1	2	3	Borre
4	5	6	
7	8	9	
+/-	0	.	

No es aplicable

Pregunta Previa

Siguiente Pregunta

Repita la pregunta

En los últimos 3 meses, de los 3 hombres con quienes tuvo sexo anal durante el sexo grupal, ¿con cuántos hombres diferentes tuvo sexo anal sin condón?

1	2	3	Borre
4	5	6	
7	8	9	
+/-	0	.	

No Sabe

Rehusó Contestar

No es aplicable

Pregunta Previa

Siguiente Pregunta

Repita la pregunta

Las siguientes tres preguntas indagarán sobre el estado de VIH (desconocido, VIH positivo o VIH negativo) de los 3 hombres con quienes tuvo sexo anal sin usar condón durante sexo grupal en los últimos 3 meses. El número total de parejas que usted ingrese en las proximas tres preguntas se debe sumar a 3. Si ninguna de estas 3 parejas se puede ser descrita con una de las categorias del estatus de VIH, por favor ingresa 0 para esa categoría.

No Sabe

Rehusó Contestar

No es aplicable

Pregunta Previa

Siguiente Pregunta

Repita la pregunta

¿Cuántos de estos 3 hombres con quienes tuvo sexo anal sin usar condón durante sexo grupal fueron parejas cuyo estado de VIH desconocía?

No Sabe

Rehusó Contestar

1 2 3

Borre

No es aplicable

4 5 6

Pregunta Previa

7 8 9

Siguiente Pregunta

+/- 0 .

Repita la pregunta

¿Cuántos de estos 3 hombres con quienes tuvo sexo anal sin usar condón durante sexo grupal fueron VIH positivo?

No Sabe

Rehusó Contestar

1	2	3	Borre
4	5	6	
7	8	9	
+/-	0	.	

No es aplicable

Pregunta Previa

Siguiente Pregunta

Repita la pregunta

¿Cuántos de estos 3 hombres con quienes tuvo sexo anal sin usar condón durante sexo grupal fueron VIH negativo?

No Sabe

Rehusó Contestar

1 2 3

Borre

No es aplicable

4 5 6

Pregunta Previa

7 8 9

Siguiente Pregunta

+/- 0 .

Repita la pregunta

La siguiente sección indagará sobre los 5 hombres con quienes tuvo sexo anal.

No Sabe

Rehusó Contestar

No es aplicable

Pregunta Previa

Siguiente Pregunta

Repita la pregunta

Por favor ingrese un apodo o iniciales para los 3 hombres más recientes con quienes tuvo sexo anal desde 11/11/2017. No queremos saber los nombres de sus parejas. Elija un apodo o iniciales que recuerde y que le ayuden a identificar mejor a dicha persona.

No Sabe

Rehusó Contestar

No es aplicable

Pregunta Previa

Siguiente Pregunta

Repita la pregunta

Por favor ingrese un apodo o iniciales para la pareja 1:
Si no desea ingresar un apodo o iniciales, ingrese "pareja 1."

A	B	C	D	E	F	G	Borre
H	I	J	K	L	M	N	Retroceda
O	P	Q	R	S	T	U	Alt
V	W	X	Y	Z			

No Sabe

Rehusó Contestar

No es aplicable

Pregunta Previa

Siguiente Pregunta

Repita la pregunta

Por favor ingrese un apodo o iniciales para la pareja 2:
Si no desea ingresar un apodo o iniciales, ingrese "pareja 2."

A	B	C	D	E	F	G	Borre
H	I	J	K	L	M	N	Retroceda
O	P	Q	R	S	T	U	Alt
V	W	X	Y	Z			

No Sabe

Rehusó Contestar

No es aplicable

Pregunta Previa

Siguiente Pregunta

Repita la pregunta

Por favor ingrese un apodo o iniciales para la pareja 3:
Si no desea ingresar un apodo o iniciales, ingrese "pareja 3."

A	B	C	D	E	F	G	Borre
H	I	J	K	L	M	N	Retroceda
O	P	Q	R	S	T	U	Alt
V	W	X	Y	Z			

No Sabe

Rehusó Contestar

No es aplicable

Pregunta Previa

Siguiente Pregunta

Repita la pregunta

Ahora vamos a preguntar si tuvo sexo con cada una de estas parejas durante el mes pasado, el mes anterior (entre 12/11/2017 y 01/10/2018) y el mes anterior (entre 11/11/2017 y 12/11/2017).

No Sabe

Rehusó Contestar

No es aplicable

Pregunta Previa

Siguiente Pregunta

Repita la pregunta

¿Usted y p1 tuvieron sexo el mes pasado, es decir desde 01/10/2018?

NO

Sí

No Sabe

Rehusó Contestar

No es aplicable

Pregunta Previa

Siguiente Pregunta

Repita la pregunta

¿Usted y p1 tuvieron sexo hace 2 meses, es decir entre 12/11/2017 y 01/10/2018?

NO

Sí

No Sabe

Rehusó Contestar

No es aplicable

Pregunta Previa

Siguiente Pregunta

Repita la pregunta

¿Usted y p1 tuvieron sexo hace 3 meses, es decir entre 11/11/2017 y 12/11/2017?

No Sabe

Rehusó Contestar

No es aplicable

NO

Sí

Pregunta Previa

Siguiente Pregunta

Repita la pregunta

¿Usted y p2 tuvieron sexo el mes pasado, es decir desde 01/10/2018?

No Sabe

Rehusó Contestar

No es aplicable

NO

Sí

Pregunta Previa

Siguiente Pregunta

Repita la pregunta

¿Usted y P2 tuvieron sexo hace 2 meses, es decir entre 12/30/2017 y 01/29/2018?

NO

Sí

No Sabe

Rehusó Contestar

No es aplicable

Pregunta Previa

Siguiente Pregunta

Repita la pregunta

¿Usted y p2 tuvieron sexo hace 3 meses, es decir entre 11/11/2017 y 12/11/2017?

No Sabe

Rehusó Contestar

No es aplicable

NO

Sí

Pregunta Previa

Siguiente Pregunta

Repita la pregunta

¿Usted y p3 tuvieron sexo el mes pasado, es decir desde 01/10/2018?

No Sabe

Rehusó Contestar

No es aplicable

NO

Sí

Pregunta Previa

Siguiente Pregunta

Repita la pregunta

¿Usted y p3 tuvieron sexo hace 2 meses, es decir entre 12/11/2017 y 01/10/2018?

No Sabe

Rehusó Contestar

No es aplicable

NO

Sí

Pregunta Previa

Siguiente Pregunta

Repita la pregunta

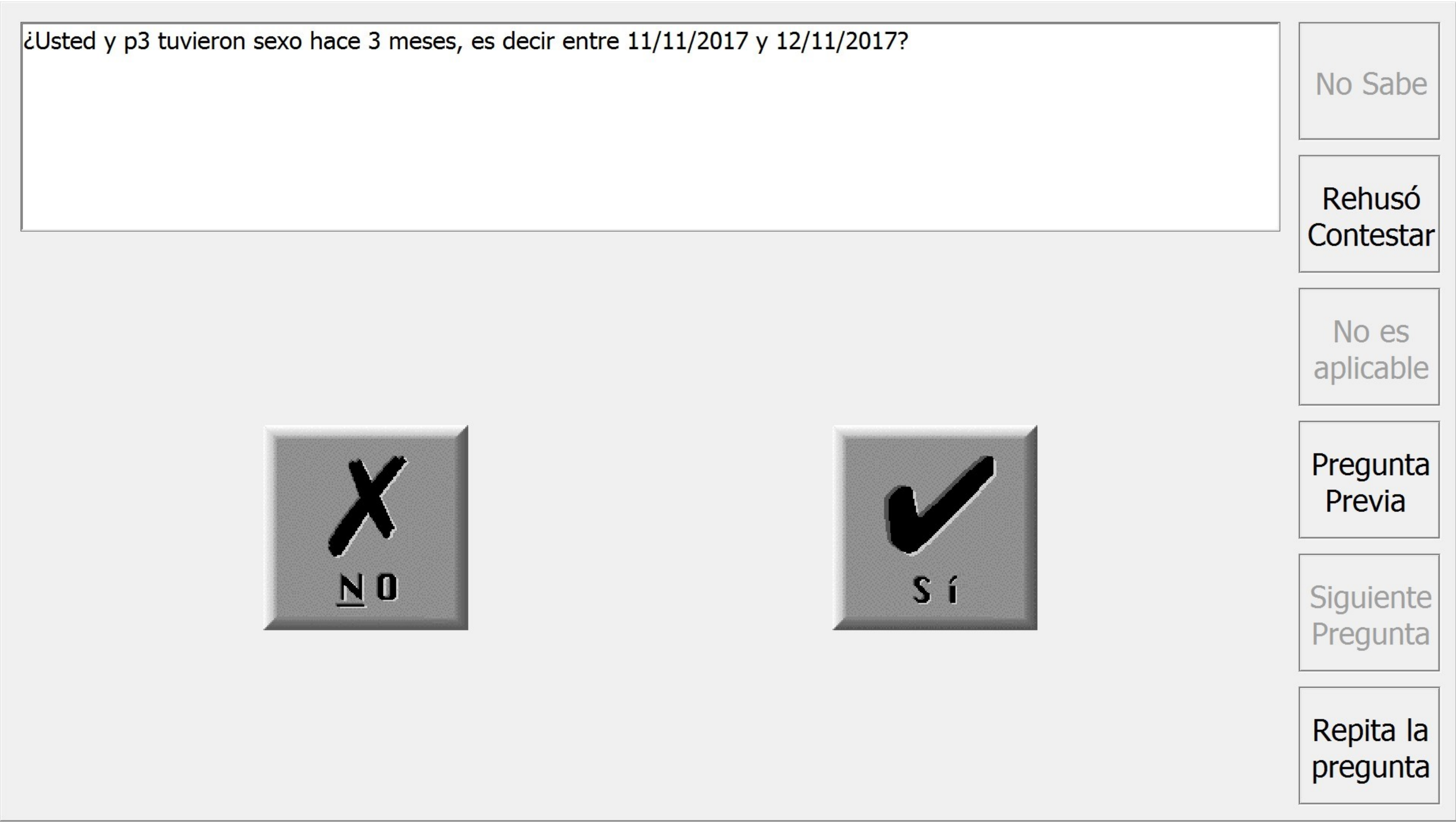
¿Usted y p3 tuvieron sexo hace 3 meses, es decir entre 11/11/2017 y 12/11/2017?
No Sabe
Rehusó Contestar
No es aplicable
Pregunta Previa
Siguiente Pregunta
Repita la pregunta
NO
Sí

Usted indicó que tuvo sexo con ambos p1 y p2 en el mismo mes. ¿Cuál de estas afirmaciones es la más cercana a la realidad?

La última vez tuve sexo con p2 antes de tener sexo con p1

La última vez tuve sexo con p1 antes de tener sexo con p2

Estuve teniendo sexo con p1 y p2 durante el mismo período

No Sabe

Rehusó Contestar

No es aplicable

Pregunta Previa

Siguiente Pregunta

Repita la pregunta

Usted indicó que tuvo sexo con ambos p1 y p3 en el mismo mes. ¿Cuál de estas afirmaciones es la más cercana a la realidad?

No Sabe

Rehusó Contestar

La última vez tuve sexo con p3 antes de tener sexo con p1

No es aplicable

La última vez tuve sexo con p1 antes de tener sexo con p3

Pregunta Previa

Siguiente Pregunta

Estuve teniendo sexo con p1 y p3 durante el mismo período

Repita la pregunta

Usted indicó que tuvo sexo con ambos p2 y p3 en el mismo mes. ¿Cuál de estas afirmaciones es la más cercana a la realidad?

No Sabe

Rehusó Contestar

La última vez tuve sexo con p3 antes de tener sexo con p2

No es aplicable

La última vez tuve sexo con p2 antes de tener sexo con p3

Pregunta Previa

Siguiente Pregunta

Estuve teniendo sexo con p2 y p3 durante el mismo período

Repita la pregunta

Gracias por contarnos sobre los meses en los que tuvo sexo con sus parejas. En la próxima página le haremos algunas preguntas para ayudarnos a comprender mejor lo que acaba de decirnos sobre sus parejas sexuales. Haga clic en Siguiente Pregunta para continuar.

No Sabe

Rehusó Contestar

No es aplicable

Pregunta Previa

Siguiente Pregunta

Repita la pregunta

¿Tuvo sexo con p1 una vez o más de una vez durante los últimos 3 meses?

No Sabe

Rehusó Contestar

Una vez

No es aplicable

Pregunta Previa

Más de una vez

Siguiente Pregunta

Repita la pregunta

¿Recuerda la fecha exacta en la que tuvo sexo anal por primera vez con p1?

No Sabe

Rehusó Contestar

No es aplicable

NO

Sí

Pregunta Previa

Siguiente Pregunta

Repita la pregunta

¿Cuándo tuvo sexo anal por primera vez con p1?
Puede ingresar solo el mes y el año, pero si conoce la fecha exacta, ingrésela. Si la primera vez que tuvo sexo con p1 fue hace más de 3 meses; aún nos gustaría saber la primera vez que tuvo sexo anal con él.

Año:	<<	<		>	>>	
Mes:	<<	<		>	>>	
Día:	<<	<		>	>>	

No Sabe

Rehusó Contestar

No es aplicable

Pregunta Previa

Siguiente Pregunta

Repita la pregunta

¿Hace cuántos años aproximadamente tuvo sexo por la primera vez con P1?

No Sabe

Rehusó Contestar

1	2	3	Borre
4	5	6	
7	8	9	
+/-	0	.	

No es aplicable

Pregunta Previa

Siguiente Pregunta

Repita la pregunta

Piense en el momento en el que tuvo sexo por primera vez con P1. Tal vez tuvo sexo durante una época especial del año, como su cumpleaños o un día festivo como el 4 de julio o Halloween. Tal vez pueda recordar que hacía calor afuera o después de un viaje que hizo. En función de lo que pueda recordar, intente seleccionar en qué momento durante [año del primer sexo] tuvo sexo por primera vez con P1:

Enero - marzo

Abril - junio

Julio - septiembre

Octubre - diciembre

No Sabe

Rehusó Contestar

No es aplicable

Pregunta Previa

Siguiente Pregunta

Repita la pregunta

¿Cuál es la fecha de la última vez que tuvo sexo anal con p1? Si recuerda la fecha exacta, ingrésela; sin embargo, si no la recuerda, indíquenos el mes y el año (puede dejar la sección del día en blanco si es necesario).

Año: << < [ ] > >>

Mes: << < [ ] > >>

Día: << < [ ] > >>

No Sabe

Rehusó Contestar

No es aplicable

Pregunta Previa

Siguiente Pregunta

Repita la pregunta

¿Recuerda la fecha exacta en la que tuvo sexo anal con P1?

No Sabe

Rehusó Contestar

No es aplicable

NO

Sí

Pregunta Previa

Siguiente Pregunta

Repita la pregunta

¿Cuándo tuvo sexo anal con P1?

Año:	<<	<		>	>>
Mes:	<<	<		>	>>
Día:	<<	<		>	>>

No Sabe

Rehusó Contestar

No es aplicable

Pregunta Previa

Siguiente Pregunta

Repita la pregunta

¿Cuál de las siguientes afirmaciones sobre la edad de p1 está más cerca de la verdad?

No Sabe

Rehusó Contestar

No es aplicable

Pregunta Previa

Siguiente Pregunta

Repita la pregunta

Él es más de 10 años menor que yo

Él es de 2 a 10 años menor que yo

Él es dentro de un año de mi edad

Él es de 2 a 10 años mayor que yo

Él es más de 10 años mayor que yo

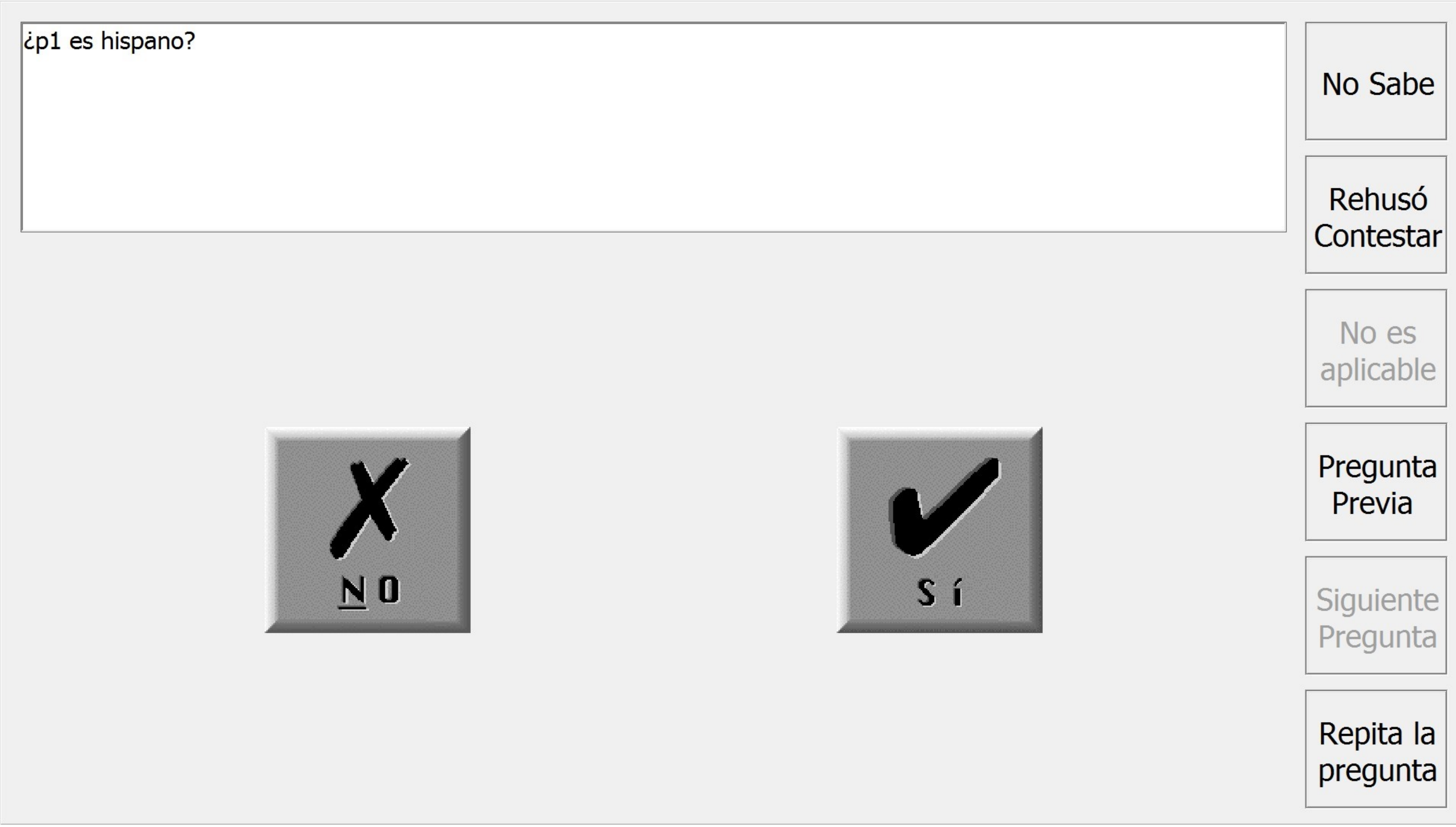
¿p1 es hispano?
NO
Sí
No Sabe
Rehusó Contestar
No es aplicable
Pregunta Previa
Siguiente Pregunta
Repita la pregunta

¿Cuál es la raza de p1? Marque todo lo que corresponda.

Indio americano o nativo de Alaska

Asiático

Negro o afroamericano

Nativo de Hawái u otra isla del Pacífico

Blanco

No Sabe

Rehusó Contestar

No es aplicable

Pregunta Previa

Siguiente Pregunta

Repita la pregunta

¿Es/fue p1 alguien con quien se siente o con quien se sintió comprometido (alguien a quien podría llamar su novio, pareja, compañero de vida o esposo)?

NO

Sí

No Sabe

Rehusó Contestar

No es aplicable

Pregunta Previa

Siguiente Pregunta

Repita la pregunta

Si tuviera que describir mejor el tipo de pareja sexual que es/fue p1,¿cuál de los siguientes elegiría? Alguien...

No Sabe

Rehusó Contestar

quien es una pareja sexual primaria

con quien usted tuvo contacto sexual solo 1 vez, pero podría volver a encontrarlo si fuera necesario

No es aplicable

con quien tiene sexo regularmente, pero que no es una pareja principal o primaria

a quien nunca había conocido antes de haber tenido contacto sexual y nunca planea volver a ver

Pregunta Previa

con quien ha tenido contacto sexual más de una vez, pero no regularmente y con quien normalmente socializa

con quien tuvo sexo por dinero u otros bienes o alguien que tuvo sexo con usted por dinero u otros bienes

Siguiente Pregunta

con quien ha tenido contacto sexual más de una vez, pero no regularmentey con quien normalmente no socializa

Repita la pregunta

¿Dónde conoció por primera vez a p1 ?

A través de amigos	En la iglesia	En la calle	Fiesta sexual privada
Escuela o trabajo	A través de un anuncio personal en un periódico	Librería para adultos	Club deportivo o gimnasio
En el internet	En una línea de chat telefónico o línea de citas	Casa de sexo	Vacación o crucero
Aplicación de teléfono	Bar/club	Club sexual	Organización social
Fiesta de circuito o Rave	Zona de levante	Complejo vacacional sexual	Otro

No Sabe

Rehusó Contestar

No es aplicable

Pregunta Previa

Siguiente Pregunta

Repita la pregunta

Usted respondió otro. Por favor usa el teclado de la computador para ingresar adonde conoció a p1 por la primera vez.

A	B	C	D	E	F	G	Borre
H	I	J	K	L	M	N	Retroceda
O	P	Q	R	S	T	U	Alt
V	W	X	Y	Z			

No Sabe

Rehusó Contestar

No es aplicable

Pregunta Previa

Siguiente Pregunta

Repita la pregunta

¿A través de qué servicio del internet conoció por primera vez a p1?

Facebook	Manhunt	Friendster
CraigsList	OKCupid	MySpace
Adam4Adam	GuySpy	Otro
BarebackRT	FindFred	

No Sabe

Rehusó Contestar

No es aplicable

Pregunta Previa

Siguiente Pregunta

Repita la pregunta

Usted respondió otro. Por favor usa el teclado de la computador para ingresar el servicio del internet a traves de que conoció p1 por la primera vez.

A	B	C	D	E	F	G	Borre
H	I	J	K	L	M	N	Retroceda
O	P	Q	R	S	T	U	Alt
V	W	X	Y	Z			

No Sabe

Rehusó Contestar

No es aplicable

Pregunta Previa

Siguiente Pregunta

Repita la pregunta

¿A través de qué aplicación de teléfono conoció por primera vez a p1?

Grindr	Adam4Adam	BoyAhoy
Scruff	Growlr	Otro
Jackd	GuySpy	
Hornet	Skout	

No Sabe

Rehusó Contestar

No es aplicable

Pregunta Previa

Siguiente Pregunta

Repita la pregunta

Usted respondió otro. Por favor usa el teclado de la computador para ingresar la aplicación de teléfono a traves de que conoció p1 por la primera vez.

No Sabe

Rehusó Contestar

A	B	C	D	E	F	G	Borre
H	I	J	K	L	M	N	Retroceda
O	P	Q	R	S	T	U	Alt
V	W	X	Y	Z			

No es aplicable

Pregunta Previa

Siguiente Pregunta

Repita la pregunta

¿Compartió su estado de VIH con p1 antes de tener sexo por primera vez?

No Sabe

Rehusó Contestar

No es aplicable

NO

SÍ

Pregunta Previa

Siguiente Pregunta

Repita la pregunta

¿p1 compartió su estado de VIH con usted antes de tener sexo por primera vez?

No Sabe

Rehusó Contestar

No es aplicable

NO

Sí

Pregunta Previa

Siguiente Pregunta

Repita la pregunta

¿Cuál fue el estado de VIH de p1 en dicho momento?

No Sabe

Rehusó Contestar

VIH-negativo

No es aplicable

Pregunta Previa

VIH-positivo

Siguiente Pregunta

Repita la pregunta

Según su conocimiento, ¿cuál es el estado de VIH de p1 hoy?

VIH negativo

VIH positivo

No Sabe

Rehusó Contestar

No es aplicable

Pregunta Previa

Siguiente Pregunta

Repita la pregunta

De acuerdo con su conocimiento, ¿actualmente p1 está tomando medicamentos para evitar contraer VIH? Esto también podría conocerse como profilaxis previa a la exposición (PrEP).

No Sabe

Rehusó Contestar

No es aplicable

NO

Sí

Pregunta Previa

Siguiente Pregunta

Repita la pregunta

De acuerdo con su conocimiento, ¿actualmente P1 está tomando medicamentos para VIH?

NO

Sí

No Sabe

Rehusó Contestar

No es aplicable

Pregunta Previa

Siguiente Pregunta

Repita la pregunta

De acuerdo con su conocimiento, ¿P1 tiene una carga viral indetectable?

NO

Sí

No Sabe

Rehusó Contestar

No es aplicable

Pregunta Previa

Siguiente Pregunta

Repita la pregunta

En los últimos 3 meses (desde 11/11/2017), ¿cuántas veces tuvieron usted y p1 sexo anal?

No Sabe

Rehusó Contestar

1 2 3

Borre

No es aplicable

4 5 6

Pregunta Previa

7 8 9

Siguiente Pregunta

+/- 0 .

Repita la pregunta

Cuando tuvo sexo anal con p1 , ¿tuvo sexo anal sin usar condón o no lo usó todo el tiempo?

No Sabe

Rehusó Contestar

No es aplicable

Pregunta Previa

Siguiente Pregunta

Repita la pregunta

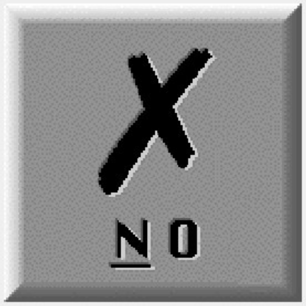

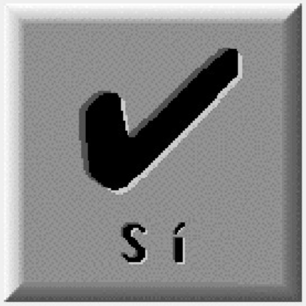

En los últimos 3 meses (desde 11/11/2017), ¿cuántas veces tuvieron usted y p1 sexo anal sin usar condón o sin usarlo todo el tiempo?

No Sabe

Rehusó Contestar

1	2	3	Borre
4	5	6	
7	8	9	
+/-	0	.	

No es aplicable

Pregunta Previa

Siguiente Pregunta

Repita la pregunta

En los últimos 3 meses (desde 11/11/2017), cuando usted y p1 tuvieron sexo anal sin condón, ¿usted estuvo arriba (su pene en el trasero de él), abajo (su pene en el trasero de usted) o ambos? Marque solo una respuesta.

Solo abajo

Solo arriba

Tanto arriba como abajo

No Sabe

Rehusó Contestar

No es aplicable

Pregunta Previa

Siguiente Pregunta

Repita la pregunta

En los últimos 3 meses (desde 11/11/2017), durante el tiempo en el que tuvo sexo con p1, ¿p1 tuvo sexo con alguien más?

NO

Sí

No Sabe

Rehusó Contestar

No es aplicable

Pregunta Previa

Siguiente Pregunta

Repita la pregunta

¿ p1 tuvo algún diagnóstico de ETS en los últimos 3 meses, es decir desde 11/11/2017)?

NO

Sí

No Sabe

Rehusó Contestar

No es aplicable

Pregunta Previa

Siguiente Pregunta

Repita la pregunta

Las siguientes preguntas indagarán sobre las veces que usted y p1 tuvieron sexo con al menos otro hombre juntos en el mismo encuentro. Para estas preguntas, un trío implica sexo con usted, p1 y otro hombre (un total de 3 hombres). El sexo grupal implica sexo con usted, p1 y al menos otros 2 hombres (un total de 4 hombres o más). Cuando la pregunta indague sobre la cantidad de parejas que tuvo durante un trío o sexo grupal, escriba el número total de parejas diferentes que tuvo en todos los encuentros.

No Sabe

Rehusó Contestar

No es aplicable

Pregunta Previa

Siguiente Pregunta

Repita la pregunta

En los últimos 3 meses (desde 11/11/2017), ¿usted y p1 alguna vez tuvieron sexo con otras personas en el mismo encuentro? (Trío o sexo grupal)

No Sabe

Rehusó Contestar

No es aplicable

Pregunta Previa

Siguiente Pregunta

Repita la pregunta

NO

Sí

Las siguientes preguntas indagarán sobre las veces que usted y p1 tuvieron sexo con al menos otro hombre juntos en el mismo encuentro. Para estas preguntas, un trío implica sexo con usted, p1 y otro hombre (un total de 3 hombres). El sexo grupal implica sexo con usted, p1 y al menos otros 2 hombres (un total de 4 hombres o más). Cuando la pregunta indague sobre la cantidad de parejas que tuvo durante un trío o sexo grupal, escriba el número total de parejas diferentes que tuvo en todos los encuentros.

No Sabe

Rehusó Contestar

No es aplicable

Pregunta Previa

Siguiente Pregunta

Repita la pregunta

Piense en la última vez que tuvo un trío con p1. ¿Alguien tuvo sexo anal durante este trío?

No Sabe

Rehusó Contestar

No es aplicable

NO

Sí

Pregunta Previa

Siguiente Pregunta

Repita la pregunta

¿Alguien tuvo sexo anal sin condón durante este trío?

NO

Sí

No Sabe

Rehusó Contestar

No es aplicable

Pregunta Previa

Siguiente Pregunta

Repita la pregunta

¿Quién tuvo sexo anal sin condón el uno con el otro durante este trío? Marque todo lo que corresponda.

No Sabe

Rehusó Contestar

No es aplicable

Pregunta Previa

Siguiente Pregunta

Repita la pregunta

Tuve sexo anal sin condón con p1

Tuve sexo anal sin condón con la 3a pareja

p1 tuvo sexo anal sin condón con la 3a pareja.

¿Cuál era el estado de VIH de la 3a pareja?

VIH negativo

VIH positivo

No conocía su estado de VIH

No Sabe

Rehusó Contestar

No es aplicable

Pregunta Previa

Siguiente Pregunta

Repita la pregunta

¿Cuántas veces usted y p1 tuvieron sexo con otros 2 o más hombres en el mismo encuentro (sexo grupal) en los últimos 3 meses?

No Sabe

Rehusó Contestar

1	2	3	Borre
4	5	6	
7	8	9	
+/-	0	.	

No es aplicable

Pregunta Previa

Siguiente Pregunta

Repita la pregunta

Piense en la última vez que tuvo sexo grupal con p1 y al menos otros dos hombres. ¿Cuántos hombres participaron en este encuentro de sexo grupal? Incluya a p1, pero no a sí mismo.

No Sabe

Rehusó Contestar

1	2	3	Borre
4	5	6	
7	8	9	
+/-	0	.	

No es aplicable

Pregunta Previa

Siguiente Pregunta

Repita la pregunta

Las siguientes tres preguntas indagarán sobre el estado de VIH (desconocido, VIH positivo o VIH negativo) de los 6 hombres involucrados la última vez que tuvo sexo grupal con p1. El número total de parejas que usted ingresa en las siguientes tres preguntas se debe sumar a 6. Si ninguna de estas 6 parejas se puede ser descrita con una de las categorías del estado de VIH, por favor ingrese 0 para esa categoría

No Sabe

Rehusó Contestar

No es aplicable

Pregunta Previa

Siguiente Pregunta

Repita la pregunta

¿Cuántos de estos 6 hombres involucrados en la última vez que tuvo sexo grupal con p1 fueron parejas cuyo estado de VIH desconocía?

No Sabe

Rehusó Contestar

1	2	3	Borre
4	5	6	
7	8	9	
+/-	0	.	

No es aplicable

Pregunta Previa

Siguiente Pregunta

Repita la pregunta

¿Cuántos de estos 6 hombres involucrados en la última vez que tuvo sexo grupal con p1 fueron VIH positivos?

1	2	3	Borre
4	5	6	
7	8	9	
+/-	0	.	

No Sabe

Rehusó Contestar

No es aplicable

Pregunta Previa

Siguiente Pregunta

Repita la pregunta

¿Cuántos de estos 6 hombres involucrados en la última vez que tuvo sexo grupal con p1 fueron VIH negativos?

No Sabe

Rehusó Contestar

1 2 3

Borre

No es aplicable

4 5 6

Pregunta Previa

7 8 9

Siguiente Pregunta

+/- 0 .

Repita la pregunta

¿Tuvo sexo anal con p1 **sin usar condón** en su último encuentro de sexo grupal que incluyó p1?

No Sabe

Rehusó Contestar

No es aplicable

Pregunta Previa

Siguiente Pregunta

Repita la pregunta

NO

Sí

¿Tuvo sexo anal con la 3a pareja **sin usar condón** en su último encuentro de sexo grupal que incluyó p1?

No Sabe

Rehusó Contestar

No es aplicable

NO

Sí

Pregunta Previa

Siguiente Pregunta

Repita la pregunta

¿p1 y la 3a pareja tuvieron sexo anal **sin usar condón** en su último encuentro de sexo grupal que incluyó p1?

No Sabe

Rehusó Contestar

No es aplicable

Pregunta Previa

Siguiente Pregunta

Repita la pregunta

NO

Sí

¿Tuvo sexo anal con la 4a pareja **sin usar condón** en su último encuentro de sexo grupal que incluyó p1?

NO

SÍ

No Sabe

Rehusó Contestar

No es aplicable

Pregunta Previa

Siguiente Pregunta

Repita la pregunta

¿p1 y la 4a pareja tuvieron sexo anal **sin usar condón** en su último encuentro de sexo grupal que incluyó p1?

NO

Sí

No Sabe

Rehusó Contestar

No es aplicable

Pregunta Previa

Siguiente Pregunta

Repita la pregunta

¿La 3a pareja y la 4 a pareja tuvieron sexo anal **sin usar condón** en su último encuentro de sexo grupal que incluyó p1?

No Sabe

Rehusó Contestar

No es aplicable

NO

Sí

Pregunta Previa

Siguiente Pregunta

Repita la pregunta

¿Tuvo sexo anal con la 5a pareja **sin usar condón** en su último encuentro de sexo grupal que incluyó p1?

NO

Sí

No Sabe

Rehusó Contestar

No es aplicable

Pregunta Previa

Siguiente Pregunta

Repita la pregunta

¿p1 y la 5 a pareja tuvieron sexo anal **sin usar condón** en su último encuentro de sexo grupal que incluyó p1?

No Sabe

Rehusó Contestar

No es aplicable

Pregunta Previa

Siguiente Pregunta

Repita la pregunta

NO

Sí

¿La 3a pareja y la 5 a pareja tuvieron sexo anal **sin usar condón** en su último encuentro de sexo grupal que incluyó p1?

NO

SÍ

No Sabe

Rehusó Contestar

No es aplicable

Pregunta Previa

Siguiente Pregunta

Repita la pregunta

¿La 4a pareja y la 5 a pareja tuvieron sexo anal **sin usar condón** en su último encuentro de sexo grupal que incluyó p1?

NO

SÍ

No Sabe

Rehusó Contestar

No es aplicable

Pregunta Previa

Siguiente Pregunta

Repita la pregunta

¿Tuvo sexo con P2 una vez o más de una vez durante los últimos 3 meses?

No Sabe

Rehusó Contestar

Una vez

No es aplicable

Pregunta Previa

Más de una vez

Siguiente Pregunta

Repita la pregunta

¿Recuerda la fecha exacta en la que tuvo sexo anal por primera vez con P2?

No Sabe

Rehusó Contestar

No es aplicable

Pregunta Previa

Siguiente Pregunta

Repita la pregunta

NO

SÍ

¿Cuándo tuvo sexo anal por primera vez con P2?
Puede ingresar solo el mes y el año, pero si conoce la fecha exacta, ingrésela.
Si la primera vez que tuvo sexo con P2 fue hace más de 3 meses; aún nos gustaría saber la primera vez que tuvo sexo anal con él.

Año: << < [ ] > >>

Mes: << < [ ] > >>

Día: << < [ ] > >>

No Sabe

Rehusó Contestar

No es aplicable

Pregunta Previa

Siguiente Pregunta

Repita la pregunta

¿Hace cuántos años aproximadamente tuvo sexo por la primera vez con P2?

No Sabe

Rehusó Contestar

1	2	3	Borre
4	5	6	
7	8	9	
+/-	0	.	

No es aplicable

Pregunta Previa

Siguiente Pregunta

Repita la pregunta

Piense en el momento en el que tuvo sexo por primera vez con P2. Tal vez tuvo sexo durante una época especial del año, como su cumpleaños o un día festivo como el 4 de julio o Halloween. Tal vez pueda recordar que hacía calor afuera o después de un viaje que hizo. En función de lo que pueda recordar, intente seleccionar en qué momento durante [año del primer sexo] tuvo sexo por primera vez con P2:

No Sabe

Rehusó Contestar

Enero - marzo

No es aplicable

Abril - junio

Pregunta Previa

Julio - septiembre

Siguiente Pregunta

Octubre - diciembre

Repita la pregunta

¿Cuál es la fecha de la última vez que tuvo sexo anal con P2? Si recuerda la fecha exacta, ingrésela; sin embargo, si no la recuerda, indíquenos el mes y el año (puede dejar la sección del día en blanco si es necesario).

Año:	<<	<		>	>>	
Mes:	<<	<		>	>>	
Día:	<<	<		>	>>	

No Sabe

Rehusó Contestar

No es aplicable

Pregunta Previa

Siguiente Pregunta

Repita la pregunta

¿Recuerda la fecha exacta en la que tuvo sexo anal con P2?

No Sabe

Rehusó Contestar

No es aplicable

NO

Sí

Pregunta Previa

Siguiente Pregunta

Repita la pregunta

¿Cuándo tuvo sexo anal con P2?

Año:	<<	<		>	>>
Mes:	<<	<		>	>>
Día:	<<	<		>	>>

No Sabe

Rehusó Contestar

No es aplicable

Pregunta Previa

Siguiente Pregunta

Repita la pregunta

¿Cuál de las siguientes afirmaciones sobre la edad de P2 está más cerca de la verdad?

Él es más de 10 años menor que yo

Él es de 2 a 10 años menor que yo

Él es dentro de un año de mi edad

Él es de 2 a 10 años mayor que yo

Él es más de 10 años mayor que yo

No Sabe

Rehusó Contestar

No es aplicable

Pregunta Previa

Siguiente Pregunta

Repita la pregunta

¿P2 es hispano?

NO

Sí

No Sabe

Rehusó Contestar

No es aplicable

Pregunta Previa

Siguiente Pregunta

Repita la pregunta

¿Cuál es la raza de P2? Marque todo lo que corresponda.

No Sabe

Rehusó Contestar

Indio americano o nativo de Alaska

No es aplicable

Asiático

Pregunta Previa

Negro o afroamericano

Siguiente Pregunta

Nativo de Hawái u otra isla del Pacífico

Blanco

Repita la pregunta

¿Es/fue P2 alguien con quien se siente o con quien se sintió comprometido (alguien a quien podría llamar su novio, pareja, compañero de vida o esposo)?

NO

Sí

No Sabe

Rehusó Contestar

No es aplicable

Pregunta Previa

Siguiente Pregunta

Repita la pregunta

Si tuviera que describir mejor el tipo de pareja sexual que es/fue P2,¿cuál de los siguientes elegiría? Alguien...

quien es una pareja sexual primaria

con quien usted tuvo contacto sexual solo 1 vez, pero podría volver a encontrarlo si fuera necesario

con quien tiene sexo regularmente, pero que no es una pareja principal o primaria

a quien nunca había conocido antes de haber tenido contacto sexual y nunca planea volver a ver

con quien ha tenido contacto sexual más de una vez, pero no regularmente y con quien normalmente socializa

con quien tuvo sexo por dinero u otros bienes o alguien que tuvo sexo con usted por dinero u otros bienes

con quien ha tenido contacto sexual más de una vez, pero no regularmentey con quien normalmente no socializa

No Sabe

Rehusó Contestar

No es aplicable

Pregunta Previa

Siguiente Pregunta

Repita la pregunta

¿Dónde conoció por primera vez a P2?

A través de amigos	En la iglesia	En la calle	Fiesta sexual privada
Escuela o trabajo	A través de un anuncio personal en un periódico	Librería para adultos	Club deportivo o gimnasio
En el internet	En una línea de chat telefónico o línea de citas	Casa de sexo	Vacación o crucero
Aplicación de teléfono	Bar/club	Club sexual	Organización social
Fiesta de circuito o Rave	Zona de levante	Complejo vacacional sexual	Otro

No Sabe

Rehusó Contestar

No es aplicable

Pregunta Previa

Siguiente Pregunta

Repita la pregunta

Usted respondió otro. Por favor usa el teclado de la computador para ingresar adonde conoció P2 por la primera vez.

A	B	C	D	E	F	G	Borre	No Sabe
H	I	J	K	L	M	N	Retroceda	Rehusó Contestar
O	P	Q	R	S	T	U	Alt	No es aplicable
V	W	X	Y	Z				Pregunta Previa
								Siguiente Pregunta
								Repita la pregunta

¿A través de qué servicio del internet conoció por primera vez a P2?

Facebook	Manhunt	Friendster
CraigsList	OKCupid	MySpace
Adam4Adam	GuySpy	Otro
BarebackRT	FindFred	

No Sabe

Rehusó Contestar

No es aplicable

Pregunta Previa

Siguiente Pregunta

Repita la pregunta

Usted respondió otro. Por favor usa el teclado de la computador para ingresar el servicio del internet a traves de que conoció P2 por la primera vez.

A	B	C	D	E	F	G	Borre
H	I	J	K	L	M	N	Retroceda
O	P	Q	R	S	T	U	Alt
V	W	X	Y	Z			

No Sabe

Rehusó Contestar

No es aplicable

Pregunta Previa

Siguiente Pregunta

Repita la pregunta

¿A través de qué aplicación de teléfono conoció por primera vez a P2?

Grindr	Adam4Adam	BoyAhoy
Scruff	Growlr	Otro
Jackd	GuySpy	
Hornet	Skout	

No Sabe

Rehusó Contestar

No es aplicable

Pregunta Previa

Siguiente Pregunta

Repita la pregunta

Usted respondió otro. Por favor usa el teclado de la computador para ingresar la aplicación de teléfono a traves de que conoció P2 por la primera vez.

No Sabe

Rehusó Contestar

No es aplicable

Pregunta Previa

Siguiente Pregunta

Repita la pregunta

A	B	C	D	E	F	G	Borre
H	I	J	K	L	M	N	Retroceda
O	P	Q	R	S	T	U	Alt
V	W	X	Y	Z			

¿Compartió su estado de VIH con P2 antes de tener sexo por primera vez?

No Sabe

Rehusó Contestar

No es aplicable

Pregunta Previa

Siguiente Pregunta

Repita la pregunta

NO

Sí

¿P2 compartió su estado de VIH con usted antes de tener sexo por primera vez?

No Sabe

Rehusó Contestar

No es aplicable

NO

Sí

Pregunta Previa

Siguiente Pregunta

Repita la pregunta

¿Cuál fue el estado de VIH de P2 en dicho momento?

No Sabe

Rehusó Contestar

VIH negativo

No es aplicable

Pregunta Previa

Siguiente Pregunta

VIH positivo

Repita la pregunta

Según su conocimiento, ¿cuál es el estado de VIH de P2 hoy?

No Sabe

Rehusó Contestar

No es aplicable

Pregunta Previa

Siguiente Pregunta

Repita la pregunta

VIH negativo

VIH positivo

De acuerdo con su conocimiento, ¿actualmente P2 está tomando medicamentos para evitar contraer VIH? Esto también podría conocerse como profilaxis previa a la exposición (PrEP).

NO

SÍ

No Sabe

Rehusó Contestar

No es aplicable

Pregunta Previa

Siguiente Pregunta

Repita la pregunta

De acuerdo con su conocimiento, ¿actualmente P2 está tomando medicamentos para VIH?

No Sabe

Rehusó Contestar

No es aplicable

Pregunta Previa

Siguiente Pregunta

Repita la pregunta

NO

Sí

De acuerdo con su conocimiento, ¿P2 tiene una carga viral indetectable?

No Sabe

Rehusó Contestar

No es aplicable

Pregunta Previa

Siguiente Pregunta

Repita la pregunta

NO

Sí

En los últimos 3 meses (desde 12/01/2017), ¿cuántas veces tuvieron usted y P2 sexo anal?

No Sabe

Rehusó Contestar

1 2 3 Borre

No es aplicable

4 5 6

Pregunta Previa

7 8 9

Siguiente Pregunta

+/- 0 .

Repita la pregunta

Cuando tuvo sexo anal con P2 , ¿tuvo sexo anal sin usar condón o no lo usó todo el tiempo?

No Sabe

Rehusó Contestar

No es aplicable

NO

Sí

Pregunta Previa

Siguiente Pregunta

Repita la pregunta

En los últimos 3 meses (desde 12/01/2017), ¿cuántas veces tuvieron usted y P2 sexo anal sin usar condón o sin usarlo todo el tiempo?

No Sabe

Rehusó Contestar

1	2	3	Borre
4	5	6	
7	8	9	
+/-	0	.	

No es aplicable

Pregunta Previa

Siguiente Pregunta

Repita la pregunta

En los últimos 3 meses (desde 12/01/2017), cuando usted y P2 tuvieron sexo anal sin condón, ¿usted estuvo arriba (su pene en el trasero de él), abajo (su pene en el trasero de usted) o ambos? Marque solo una respuesta.

Solo abajo

Solo arriba

Tanto arriba como abajo

No Sabe

Rehusó Contestar

No es aplicable

Pregunta Previa

Siguiente Pregunta

Repita la pregunta

En los últimos 3 meses (desde 12/01/2017), durante el tiempo en el que tuvo sexo con P2, ¿P2 tuvo sexo con alguien más?

No Sabe

Rehusó Contestar

No es aplicable

NO

Sí

Pregunta Previa

Siguiente Pregunta

Repita la pregunta

¿P2 tuvo algún diagnóstico de ETS en los últimos 3 meses, es decir desde 12/01/2017)?

NO

Sí

No Sabe

Rehusó Contestar

No es aplicable

Pregunta Previa

Siguiente Pregunta

Repita la pregunta

Las siguientes preguntas indagarán sobre las veces que usted y P2 tuvieron sexo con al menos otro hombre juntos en el mismo encuentro. Para estas preguntas, un trío implica sexo con usted, P2 y otro hombre (un total de 3 hombres). El sexo grupal implica sexo con usted, P2 y al menos otros 2 hombres (un total de 4 hombres o más). Cuando la pregunta indague sobre la cantidad de parejas que tuvo durante un trío o sexo grupal, escriba el número total de parejas diferentes que tuvo en todos los encuentros.

No Sabe

Rehusó Contestar

No es aplicable

Pregunta Previa

Siguiente Pregunta

Repita la pregunta

En los últimos 3 meses (desde 12/01/2017), ¿usted y P2 alguna vez tuvieron sexo con otras personas en el mismo encuentro? (Trío o sexo grupal)

NO

Sí

No Sabe

Rehusó Contestar

No es aplicable

Pregunta Previa

Siguiente Pregunta

Repita la pregunta

¿Cuántas veces usted y P2 tuvieron sexo solo con otro hombre en el mismo encuentro (un trío) en los últimos 3 meses?

1	2	3	Borre
4	5	6	
7	8	9	
+/-	0	.	

No Sabe

Rehusó Contestar

No es aplicable

Pregunta Previa

Siguiente Pregunta

Repita la pregunta

Piense en la última vez que tuvo un trío con P2. ¿Alguien tuvo sexo anal durante este trío?

NO

Sí

No Sabe

Rehusó Contestar

No es aplicable

Pregunta Previa

Siguiente Pregunta

Repita la pregunta

¿Alguien tuvo sexo anal sin condón durante este trío?

No Sabe

Rehusó Contestar

No es aplicable

Pregunta Previa

Siguiente Pregunta

Repita la pregunta

NO

Sí

¿Quién tuvo sexo anal sin condón el uno con el otro durante este trío? Marque todo lo que corresponda.

No Sabe

Rehusó Contestar

No es aplicable

Pregunta Previa

Siguiente Pregunta

Repita la pregunta

Tuve sexo anal sin condón con P2

Tuve sexo anal sin condón con la 3a pareja

P2 tuvo sexo anal sin condón con la 3a pareja.

¿Cuál era el estado de VIH de la 3a pareja?

VIH negativo

VIH positivo

No conocía su estado de VIH

No Sabe

Rehusó Contestar

No es aplicable

Pregunta Previa

Siguiente Pregunta

Repita la pregunta

¿Cuántas veces usted y P2 tuvieron sexo con otros 2 o más hombres en el mismo encuentro (sexo grupal) en los últimos 3 meses?

1	2	3	Borre
4	5	6	
7	8	9	
+/-	0	.	

No Sabe

Rehusó Contestar

No es aplicable

Pregunta Previa

Siguiente Pregunta

Repita la pregunta

Piense en la última vez que tuvo sexo grupal con P2 y al menos otros dos hombres. ¿Cuántos hombres participaron en este encuentro de sexo grupal? Incluya a P2, pero no a sí mismo.

No Sabe

Rehusó Contestar

1 2 3

Borre

No es aplicable

4 5 6

Pregunta Previa

7 8 9

Siguiente Pregunta

+/- 0 .

Repita la pregunta

Las siguientes tres preguntas indagarán sobre el estado de VIH (desconocido, VIH positivo o VIH negativo) de los 6 hombres involucrados la última vez que tuvo sexo grupal con P2. El número total de parejas que usted ingresa en las siguientes tres preguntas se debe sumar a 6. Si ninguna de estas 6 parejas se puede ser descrita con una de las categorías del estado de VIH, por favor ingrese 0 para esa categoría

No Sabe

Rehusó Contestar

No es aplicable

Pregunta Previa

Siguiente Pregunta

Repita la pregunta

¿Cuántos de estos 6 hombres involucrados en la última vez que tuvo sexo grupal con P2 fueron parejas cuyo estado de VIH desconocía?

1	2	3	Borre
4	5	6	
7	8	9	
+/-	0	.	

No Sabe

Rehusó Contestar

No es aplicable

Pregunta Previa

Siguiente Pregunta

Repita la pregunta

¿Cuántos de estos 6 hombres involucrados en la última vez que tuvo sexo grupal con P2 fueron VIH positivos?

No Sabe

Rehusó Contestar

1	2	3	Borre
4	5	6	
7	8	9	
+/-	0	.	

No es aplicable

Pregunta Previa

Siguiente Pregunta

Repita la pregunta

¿Cuántos de estos 6 hombres involucrados en la última vez que tuvo sexo grupal con P2 fueron VIH negativos?

No Sabe

Rehusó Contestar

1	2	3	Borre
4	5	6	
7	8	9	
+/-	0	.	

No es aplicable

Pregunta Previa

Siguiente Pregunta

Repita la pregunta

¿Tuvo sexo anal con P2 **sin usar condón** en su último encuentro de sexo grupal que incluyó P2?

No Sabe

Rehusó Contestar

No es aplicable

NO

Sí

Pregunta Previa

Siguiente Pregunta

Repita la pregunta

¿Tuvo sexo anal con la 3a pareja **sin usar condón** en su último encuentro de sexo grupal que incluyó P2?

No Sabe

Rehusó Contestar

No es aplicable

Pregunta Previa

Siguiente Pregunta

Repita la pregunta

NO

Sí

¿P2 y la 3a pareja tuvieron sexo anal **sin usar condón** en su último encuentro de sexo grupal que incluyó P2?

No Sabe

Rehusó Contestar

No es aplicable

Pregunta Previa

Siguiente Pregunta

Repita la pregunta

NO

Sí

¿Tuvo sexo anal con la 4a pareja **sin usar condón** en su último encuentro de sexo grupal que incluyó P2?

No Sabe

Rehusó Contestar

No es aplicable

NO

Sí

Pregunta Previa

Siguiente Pregunta

Repita la pregunta

¿P2 y la 4a pareja tuvieron sexo anal **sin usar condón** en su último encuentro de sexo grupal que incluyó P2?

NO

Sí

No Sabe

Rehusó Contestar

No es aplicable

Pregunta Previa

Siguiente Pregunta

Repita la pregunta

¿La 3a pareja y la 4 a pareja tuvieron sexo anal **sin usar condón** en su último encuentro de sexo grupal que incluyó P2?

No Sabe

Rehusó Contestar

No es aplicable

Pregunta Previa

Siguiente Pregunta

Repita la pregunta

NO

Sí

¿Tuvo sexo anal con la 5a pareja **sin usar condón** en su último encuentro de sexo grupal que incluyó P2?

No Sabe

Rehusó Contestar

No es aplicable

NO

Sí

Pregunta Previa

Siguiente Pregunta

Repita la pregunta

¿P2 y la 5 a pareja tuvieron sexo anal **sin usar condón** en su último encuentro de sexo grupal que incluyó P2?

NO

Sí

No Sabe

Rehusó Contestar

No es aplicable

Pregunta Previa

Siguiente Pregunta

Repita la pregunta

¿La 3a pareja y la 5 a pareja tuvieron sexo anal **sin usar condón** en su último encuentro de sexo grupal que incluyó P2?

No Sabe

Rehusó Contestar

No es aplicable

NO

Sí

Pregunta Previa

Siguiente Pregunta

Repita la pregunta

¿La 4a pareja y la 5 a pareja tuvieron sexo anal **sin usar condón** en su último encuentro de sexo grupal que incluyó P2?

No Sabe

Rehusó Contestar

No es aplicable

NO

Sí

Pregunta Previa

Siguiente Pregunta

Repita la pregunta

¿Tuvo sexo con P3una vez o más de una vez durante los últimos 3 meses?

No Sabe

Rehusó Contestar

No es aplicable

Pregunta Previa

Siguiente Pregunta

Repita la pregunta

Una vez

Más de una vez

¿Recuerda la fecha exacta en la que tuvo sexo anal por primera vez con P3?

NO

Sí

No Sabe

Rehusó Contestar

No es aplicable

Pregunta Previa

Siguiente Pregunta

Repita la pregunta

¿Cuándo tuvo sexo anal por primera vez con P3?
Puede ingresar solo el mes y el año, pero si conoce la fecha exacta, ingrésela.
Si la primera vez que tuvo sexo con P3 fue hace más de 3 meses; aún nos gustaría saber la primera vez que tuvo sexo anal con él.

Año:	<<	<		>	>>	
Mes:	<<	<		>	>>	
Día:	<<	<		>	>>	

No Sabe

Rehusó Contestar

No es aplicable

Pregunta Previa

Siguiente Pregunta

Repita la pregunta

¿Hace cuántos años aproximadamente tuvo sexo por la primera vez con P3?

No Sabe

Rehusó Contestar

1	2	3	Borre
4	5	6	
7	8	9	
+/-	0	.	

No es aplicable

Pregunta Previa

Siguiente Pregunta

Repita la pregunta

Piense en el momento en el que tuvo sexo por primera vez con P3. Tal vez tuvo sexo durante una época especial del año, como su cumpleaños o un día festivo como el 4 de julio o Halloween. Tal vez pueda recordar que hacía calor afuera o después de un viaje que hizo. En función de lo que pueda recordar, intente seleccionar en qué momento durante [año del primer sexo] tuvo sexo por primera vez con P3:

Enero - marzo

Abril - junio

Julio - septiembre

Octubre - diciembre

No Sabe

Rehusó Contestar

No es aplicable

Pregunta Previa

Siguiente Pregunta

Repita la pregunta

¿Cuál es la fecha de la última vez que tuvo sexo anal con P3? Si recuerda la fecha exacta, ingrésela; sin embargo, si no la recuerda, indíquenos el mes y el año (puede dejar la sección del día en blanco si es necesario).

Año:	<<	<		>	>>
Mes:	<<	<		>	>>
Día:	<<	<		>	>>

No Sabe

Rehusó Contestar

No es aplicable

Pregunta Previa

Siguiente Pregunta

Repita la pregunta

¿Recuerda la fecha exacta en la que tuvo sexo anal con P3?

No Sabe

Rehusó Contestar

No es aplicable

Pregunta Previa

Siguiente Pregunta

Repita la pregunta

NO

Sí

¿Cuándo tuvo sexo anal con P3?

Año:	<<	<		>	>>	No Sabe
Mes:	<<	<		>	>>	Rehusó Contestar
Día:	<<	<		>	>>	No es aplicable

Pregunta Previa

Siguiente Pregunta

Repita la pregunta

¿Cuál de las siguientes afirmaciones sobre la edad de P3 está más cerca de la verdad?

No Sabe

Rehusó Contestar

Él es más de 10 años menor que yo

No es aplicable

Él es de 2 a 10 años menor que yo

Pregunta Previa

Él es dentro de un año de mi edad

Siguiente Pregunta

Él es de 2 a 10 años mayor que yo

Él es más de 10 años mayor que yo

Repita la pregunta

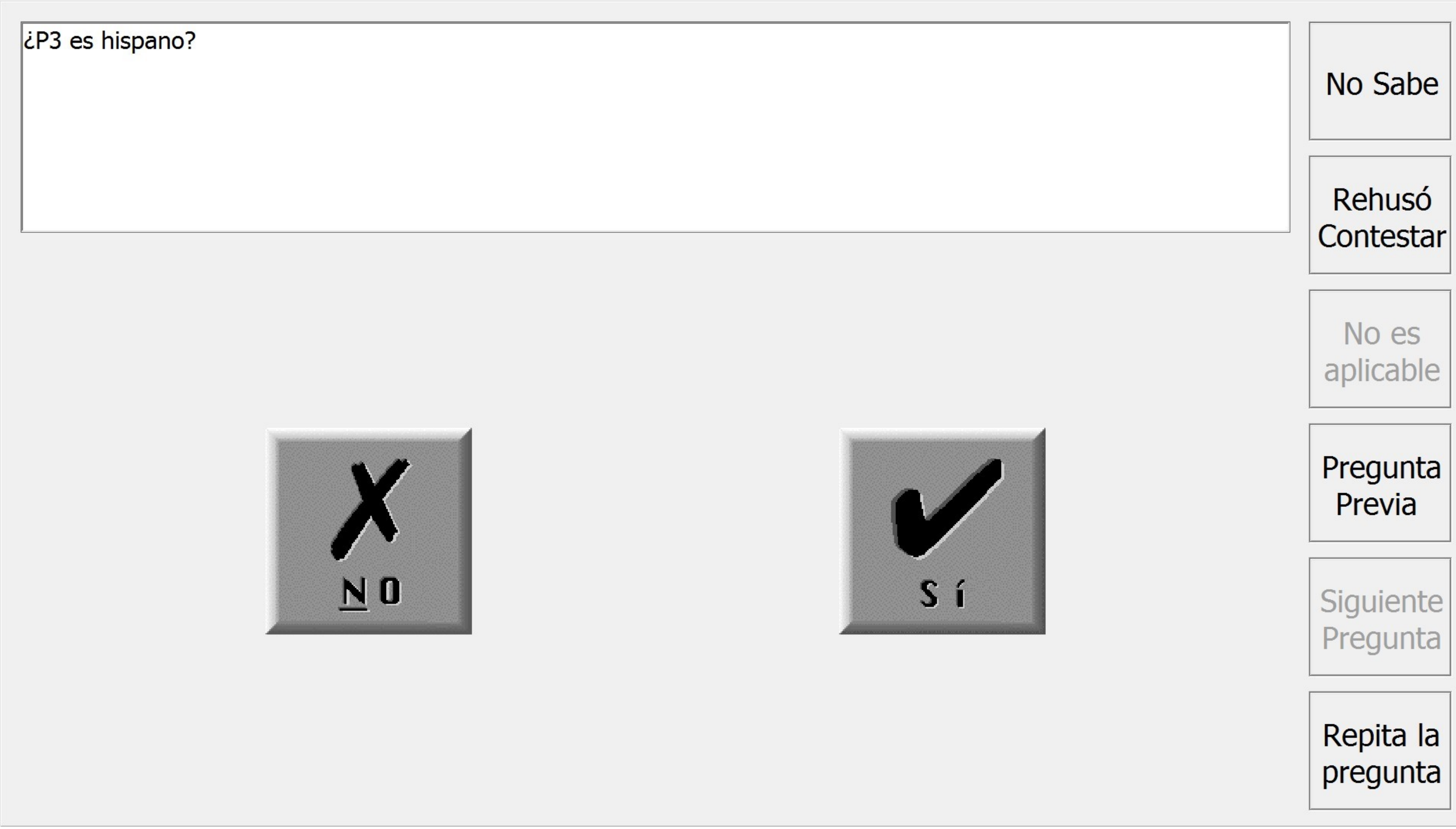
¿P3 es hispano?
No Sabe
Rehusó Contestar
No es aplicable
NO
Sí
Pregunta Previa
Siguiente Pregunta
Repita la pregunta

¿Cuál es la raza de P3? Marque todo lo que corresponda.

Indio americano o nativo de Alaska

Asiático

Negro o afroamericano

Nativo de Hawái u otra isla del Pacífico

Blanco

No Sabe

Rehusó Contestar

No es aplicable

Pregunta Previa

Siguiente Pregunta

Repita la pregunta

¿Es/fue P3 alguien con quien se siente o con quien se sintió comprometido (alguien a quien podría llamar su novio, pareja, compañero de vida o esposo)?

No Sabe

Rehusó Contestar

No es aplicable

Pregunta Previa

Siguiente Pregunta

Repita la pregunta

NO

Sí

Si tuviera que describir mejor el tipo de pareja sexual que es/fue P3,¿cuál de los siguientes elegiría? Alguien…

quien es una pareja sexual primaria

con quien usted tuvo contacto sexual solo 1 vez, pero podría volver a encontrarlo si fuera necesario

con quien tiene sexo regularmente, pero que no es una pareja principal o primaria

a quien nunca había conocido antes de haber tenido contacto sexual y nunca planea volver a ver

con quien ha tenido contacto sexual más de una vez, pero no regularmente y con quien normalmente socializa

con quien tuvo sexo por dinero u otros bienes o alguien que tuvo sexo con usted por dinero u otros bienes

con quien ha tenido contacto sexual más de una vez, pero no regularmentey con quien normalmente no socializa

No Sabe

Rehusó Contestar

No es aplicable

Pregunta Previa

Siguiente Pregunta

Repita la pregunta

¿Dónde conoció por primera vez a P3 ?

A través de amigos	En la iglesia	En la calle	Fiesta sexual privada	No Sabe
Escuela o trabajo	A través de un anuncio personal en un periódico	Librería para adultos	Club deportivo o gimnasio	Rehusó Contestar
En el internet	En una línea de chat telefónico o línea de citas	Casa de sexo	Vacación o crucero	No es aplicable
Aplicación de teléfono	Bar/club	Club sexual	Organización social	Pregunta Previa
Fiesta de circuito o Rave	Zona de levante	Complejo vacacional sexual	Otro	Siguiente Pregunta
				Repita la pregunta

Usted respondió otro. Por favor usa el teclado de la computador para ingresar adonde conoció P3 por la primera vez.

A	B	C	D	E	F	G	Borre
H	I	J	K	L	M	N	Retroceda
O	P	Q	R	S	T	U	Alt
V	W	X	Y	Z			

No Sabe

Rehusó Contestar

No es aplicable

Pregunta Previa

Siguiente Pregunta

Repita la pregunta

¿A través de qué servicio del internet conoció por primera vez a P3?

Facebook	Manhunt	Friendster	No Sabe
CraigsList	OKCupid	MySpace	Rehusó Contestar
Adam4Adam	GuySpy	Otro	No es aplicable
BarebackRT	FindFred		Pregunta Previa
			Siguiente Pregunta
			Repita la pregunta

Usted respondió otro. Por favor usa el teclado de la computador para ingresar el servicio del internet a traves de que conoció P3 por la primera vez.

A	B	C	D	E	F	G	Borre	No Sabe
H	I	J	K	L	M	N	Retroceda	Rehusó Contestar
O	P	Q	R	S	T	U	Alt	No es aplicable
V	W	X	Y	Z				Pregunta Previa
								Siguiente Pregunta
								Repita la pregunta

¿A través de qué aplicación de teléfono conoció por primera vez a P3?

Grindr	Adam4Adam	BoyAhoy
Scruff	Growlr	Otro
Jackd	GuySpy	
Hornet	Skout	

No Sabe

Rehusó Contestar

No es aplicable

Pregunta Previa

Siguiente Pregunta

Repita la pregunta

Usted respondió otro. Por favor usa el teclado de la computador para ingresar la aplicación de teléfono a traves de que conoció P3 por la primera vez.

A	B	C	D	E	F	G	Borre
H	I	J	K	L	M	N	Retroceda
O	P	Q	R	S	T	U	Alt
V	W	X	Y	Z			

No Sabe

Rehusó Contestar

No es aplicable

Pregunta Previa

Siguiente Pregunta

Repita la pregunta

¿Compartió su estado de VIH con P3 antes de tener sexo por primera vez?

No Sabe

Rehusó Contestar

No es aplicable

NO

Sí

Pregunta Previa

Siguiente Pregunta

Repita la pregunta

¿ P3 compartió su estado de VIH con usted antes de tener sexo por primera vez?

NO

Sí

No Sabe

Rehusó Contestar

No es aplicable

Pregunta Previa

Siguiente Pregunta

Repita la pregunta

¿Cuál fue el estado de VIH de P3 en dicho momento?

VIH negativo

VIH positivo

No Sabe

Rehusó Contestar

No es aplicable

Pregunta Previa

Siguiente Pregunta

Repita la pregunta

Según su conocimiento, ¿cuál es el estado de VIH de P3 hoy?

VIH negativo

VIH positivo

No Sabe

Rehusó Contestar

No es aplicable

Pregunta Previa

Siguiente Pregunta

Repita la pregunta

De acuerdo con su conocimiento, ¿actualmente P3 está tomando medicamentos para evitar contraer VIH? Esto también podría conocerse como profilaxis previa a la exposición (PrEP).

No Sabe

Rehusó Contestar

No es aplicable

Pregunta Previa

Siguiente Pregunta

Repita la pregunta

NO

SÍ

De acuerdo con su conocimiento, ¿actualmente P3 está tomando medicamentos para VIH?

No Sabe

Rehusó Contestar

No es aplicable

Pregunta Previa

Siguiente Pregunta

Repita la pregunta

NO

Sí

De acuerdo con su conocimiento, ¿P3 tiene una carga viral indetectable?

No Sabe

Rehusó Contestar

No es aplicable

NO

Sí

Pregunta Previa

Siguiente Pregunta

Repita la pregunta

En los últimos 3 meses (desde 12/01/2017), ¿cuántas veces tuvieron usted y P3 sexo anal?

1	2	3	Borre	No Sabe
4	5	6		Rehusó Contestar
7	8	9		No es aplicable
+/-	0	.		Pregunta Previa
				Siguiente Pregunta
				Repita la pregunta

Cuando tuvo sexo anal con P3 , ¿tuvo sexo anal sin usar condón o no lo usó todo el tiempo?

No Sabe

Rehusó Contestar

No es aplicable

Pregunta Previa

Siguiente Pregunta

Repita la pregunta

NO

Sí

En los últimos 3 meses (desde 12/01/2017), ¿cuántas veces tuvieron usted y P3 sexo anal sin usar condón o sin usarlo todo el tiempo?

No Sabe

Rehusó Contestar

1	2	3	Borre
4	5	6	
7	8	9	
+/-	0	.	

No es aplicable

Pregunta Previa

Siguiente Pregunta

Repita la pregunta

En los últimos 3 meses (desde 12/01/2017), cuando usted y P3 tuvieron sexo anal sin condón, ¿usted estuvo arriba (su pene en el trasero de él), abajo (su pene en el trasero de usted) o ambos? Marque solo una respuesta.

No Sabe

Rehusó Contestar

Solo abajo

No es aplicable

Pregunta Previa

Solo arriba

Siguiente Pregunta

Tanto arriba como abajo

Repita la pregunta

En los últimos 3 meses (desde 12/01/2017), durante el tiempo en el que tuvo sexo con P3, ¿P3 tuvo sexo con alguien más?

NO

Sí

No Sabe

Rehusó Contestar

No es aplicable

Pregunta Previa

Siguiente Pregunta

Repita la pregunta

¿P3 tuvo algún diagnóstico de ETS en los últimos 3 meses, es decir desde 12/01/2017)?

No Sabe

Rehusó Contestar

No es aplicable

Pregunta Previa

Siguiente Pregunta

Repita la pregunta

NO

Sí

Las siguientes preguntas indagarán sobre las veces que usted y P3 tuvieron sexo con al menos otro hombre juntos en el mismo encuentro. Para estas preguntas, un trío implica sexo con usted, P3 y otro hombre (un total de 3 hombres). El sexo grupal implica sexo con usted, P3 y al menos otros 2 hombres (un total de 4 hombres o más). Cuando la pregunta indague sobre la cantidad de parejas que tuvo durante un trío o sexo grupal, escriba el número total de parejas diferentes que tuvo en todos los encuentros.

No Sabe

Rehusó Contestar

No es aplicable

Pregunta Previa

Siguiente Pregunta

Repita la pregunta

En los últimos 3 meses (desde 12/01/2017), ¿usted y P3 alguna vez tuvieron sexo con otras personas en el mismo encuentro? (Trío o sexo grupal)

No Sabe

Rehusó Contestar

No es aplicable

Pregunta Previa

Siguiente Pregunta

Repita la pregunta

NO

Sí

¿Cuántas veces usted y P3 tuvieron sexo solo con otro hombre en el mismo encuentro (un trío) en los últimos 3 meses?

1	2	3	Borre
4	5	6	
7	8	9	
+/-	0	.	

No Sabe

Rehusó Contestar

No es aplicable

Pregunta Previa

Siguiente Pregunta

Repita la pregunta

Las siguientes preguntas indagarán sobre las veces que usted y P3 tuvieron sexo con al menos otro hombre juntos en el mismo encuentro. Para estas preguntas, un trío implica sexo con usted, P3 y otro hombre (un total de 3 hombres). El sexo grupal implica sexo con usted, P3 y al menos otros 2 hombres (un total de 4 hombres o más). Cuando la pregunta indague sobre la cantidad de parejas que tuvo durante un trío o sexo grupal, escriba el número total de parejas diferentes que tuvo en todos los encuentros.

No Sabe

Rehusó Contestar

No es aplicable

Pregunta Previa

Siguiente Pregunta

Repita la pregunta

¿Alguien tuvo sexo anal sin condón durante este trío?

No Sabe

Rehusó Contestar

No es aplicable

NO

Sí

Pregunta Previa

Siguiente Pregunta

Repita la pregunta

¿Quién tuvo sexo anal sin condón el uno con el otro durante este trío? Marque todo lo que corresponda.

Tuve sexo anal sin condón con P3

Tuve sexo anal sin condón con la 3a pareja

P3 tuvo sexo anal sin condón con la 3a pareja.

No Sabe

Rehusó Contestar

No es aplicable

Pregunta Previa

Siguiente Pregunta

Repita la pregunta

¿Cuál era el estado de VIH de la 3a pareja?

No Sabe

Rehusó Contestar

VIH negativo

No es aplicable

Pregunta Previa

VIH positivo

Siguiente Pregunta

No conocía su estado de VIH

Repita la pregunta

¿Cuántas veces usted y P3 tuvieron sexo con otros 2 o más hombres en el mismo encuentro (sexo grupal) en los últimos 3 meses?

1	2	3	Borre
4	5	6	
7	8	9	
+/-	0	.	

No Sabe

Rehusó Contestar

No es aplicable

Pregunta Previa

Siguiente Pregunta

Repita la pregunta

Piense en la última vez que tuvo sexo grupal con P3 y al menos otros dos hombres. ¿Cuántos hombres participaron en este encuentro de sexo grupal? Incluya a P3, pero no a sí mismo.

No Sabe

Rehusó Contestar

1	2	3	Borre
4	5	6	
7	8	9	
+/-	0	.	

No es aplicable

Pregunta Previa

Siguiente Pregunta

Repita la pregunta

Las siguientes tres preguntas indagarán sobre el estado de VIH (desconocido, VIH positivo o VIH negativo) de los 6 hombres involucrados la última vez que tuvo sexo grupal con P3. El número total de parejas que usted ingresa en las siguientes tres preguntas se debe sumar a 6. Si ninguna de estas 6 parejas se puede ser descrita con una de las categorías del estado de VIH, por favor ingrese 0 para esa categoría

No Sabe

Rehusó Contestar

No es aplicable

Pregunta Previa

Siguiente Pregunta

Repita la pregunta

¿Cuántos de estos 6 hombres involucrados en la última vez que tuvo sexo grupal con P3 fueron parejas cuyo estado de VIH desconocía?

No Sabe

Rehusó Contestar

1	2	3	Borre
4	5	6	
7	8	9	
+/-	0	.	

No es aplicable

Pregunta Previa

Siguiente Pregunta

Repita la pregunta

¿Cuántos de estos 6 hombres involucrados en la última vez que tuvo sexo grupal con P3 fueron VIH positivos?

1	2	3	Borre
4	5	6	
7	8	9	
+/-	0	.	

No Sabe

Rehusó Contestar

No es aplicable

Pregunta Previa

Siguiente Pregunta

Repita la pregunta

¿Cuántos de estos 6 hombres involucrados en la última vez que tuvo sexo grupal con P3 fueron VIH negativos?

No Sabe

Rehusó Contestar

1 2 3

Borre

No es aplicable

4 5 6

Pregunta Previa

7 8 9

Siguiente Pregunta

+/- 0 .

Repita la pregunta

¿Tuvo sexo anal con P3 **sin usar condón** en su último encuentro de sexo grupal que incluyó P3?

No Sabe

Rehusó Contestar

No es aplicable

NO

Sí

Pregunta Previa

Siguiente Pregunta

Repita la pregunta

¿Tuvo sexo anal con la 3a pareja **sin usar condón** en su último encuentro de sexo grupal que incluyó P3?

NO

Sí

No Sabe

Rehusó Contestar

No es aplicable

Pregunta Previa

Siguiente Pregunta

Repita la pregunta

¿P3 y la 3a pareja tuvieron sexo anal **sin usar condón** en su último encuentro de sexo grupal que incluyó P3?

No Sabe

Rehusó Contestar

No es aplicable

NO

Sí

Pregunta Previa

Siguiente Pregunta

Repita la pregunta

¿Tuvo sexo anal con la 4a pareja **sin usar condón** en su último encuentro de sexo grupal que incluyó P3?

No Sabe

Rehusó Contestar

No es aplicable

Pregunta Previa

Siguiente Pregunta

Repita la pregunta

NO

Sí

¿P3 y la 4a pareja tuvieron sexo anal **sin usar condón** en su último encuentro de sexo grupal que incluyó P3?

No Sabe

Rehusó Contestar

No es aplicable

Pregunta Previa

Siguiente Pregunta

Repita la pregunta

NO

Sí

¿La 3a pareja y la 4 a pareja tuvieron sexo anal **sin usar condón** en su último encuentro de sexo grupal que incluyó P3?

NO

Sí

No Sabe

Rehusó Contestar

No es aplicable

Pregunta Previa

Siguiente Pregunta

Repita la pregunta

¿Tuvo sexo anal con la 5a pareja **sin usar condón** en su último encuentro de sexo grupal que incluyó P3?

No Sabe

Rehusó Contestar

No es aplicable

Pregunta Previa

Siguiente Pregunta

Repita la pregunta

NO

Sí

¿P3 y la 5 a pareja tuvieron sexo anal **sin usar condón** en su último encuentro de sexo grupal que incluyó P3?

No Sabe

Rehusó Contestar

No es aplicable

Pregunta Previa

Siguiente Pregunta

Repita la pregunta

NO

Sí

¿La 3a pareja y la 5 a pareja tuvieron sexo anal **sin usar condón** en su último encuentro de sexo grupal que incluyó P3?

No Sabe

Rehusó Contestar

No es aplicable

Pregunta Previa

Siguiente Pregunta

Repita la pregunta

NO

Sí

¿La 4a pareja y la 5 a pareja tuvieron sexo anal **sin usar condón** en su último encuentro de sexo grupal que incluyó P3?

No Sabe

Rehusó Contestar

No es aplicable

Pregunta Previa

Siguiente Pregunta

Repita la pregunta

NO

Sí

¿Sabe si P1 y P2 tuvieron sexo entre sí en los últimos 3 meses o cree que probablemente sí?

NO

Sí

No Sabe

Rehusó Contestar

No es aplicable

Pregunta Previa

Siguiente Pregunta

Repita la pregunta

¿Sabe si P1 y P3 tuvieron sexo entre sí en los últimos 3 meses o cree que probablemente sí?

NO

Sí

No Sabe

Rehusó Contestar

No es aplicable

Pregunta Previa

Siguiente Pregunta

Repita la pregunta

¿Sabe si P2 y P3 tuvieron sexo entre sí en los últimos 3 meses o cree que probablemente sí?

No Sabe

Rehusó Contestar

No es aplicable

Pregunta Previa

Siguiente Pregunta

Repita la pregunta

NO

Sí
